# A mathematical model and inference method for bacterial colonization in hospital units applied to active surveillance data for carbapenem-resistant enterobacteriaceae

**DOI:** 10.1371/journal.pone.0231754

**Published:** 2020-11-12

**Authors:** Karen M. Ong, Michael S. Phillips, Charles S. Peskin

**Affiliations:** 1 New York University School of Medicine, New York, New York, United States of America; 2 Courant Institute of Mathematical Sciences, New York, New York, United States of America; University of Florida, UNITED STATES

## Abstract

Widespread use of antibiotics has resulted in an increase in antimicrobial-resistant microorganisms. Although not all bacterial contact results in infection, patients can become asymptomatically colonized, increasing the risk of infection and pathogen transmission. Consequently, many institutions have begun active surveillance, but in non-research settings, the resulting data are often incomplete and may include non-random testing, making conventional epidemiological analysis problematic. We describe a mathematical model and inference method for in-hospital bacterial colonization and transmission of carbapenem-resistant Enterobacteriaceae that is tailored for analysis of active surveillance data with incomplete observations. The model and inference method make use of the full detailed state of the hospital unit, which takes into account the colonization status of each individual in the unit and not only the number of colonized patients at any given time. The inference method computes the *exact* likelihood of all possible histories consistent with partial observations (despite the exponential increase in possible states that can make likelihood calculation intractable for large hospital units), includes techniques to improve computational efficiency, is tested by computer simulation, and is applied to active surveillance data from a 13-bed rehabilitation unit in New York City. The inference method for exact likelihood calculation is applicable to other Markov models incorporating incomplete observations. The parameters that we identify are the patient–patient transmission rate, pre-existing colonization probability, and prior-to-new-patient transmission probability. Besides identifying the parameters, we predict the effects on the total prevalence (0.07 of the total colonized patient-days) of changing the parameters and estimate the increase in total prevalence attributable to patient–patient transmission (0.02) above the baseline pre-existing colonization (0.05). Simulations with a colonized versus uncolonized long-stay patient had 44% higher total prevalence, suggesting that the long-stay patient may have been a reservoir of transmission. High-priority interventions may include isolation of incoming colonized patients and repeated screening of long-stay patients.

## Introduction

Carbapenem-resistant Enterobacteriaceae (CRE) are a rising global health threat [1–7]. CRE are a family of antibiotic-resistant, gram-negative enteric bacteria [8] that harbor resistance to many “last line” treatment drugs [1] and include human pathogens such as *Klebsiella pneumoniae*, *Enterobacter cloacae*, and *Escherichia coli* [8]. The unusual combination of pathogenicity [9] and antimicrobial resistance [1] of CRE renders it a major cause of morbidity and mortality in hospitalized patients [9] and, increasingly, otherwise healthy hosts [10]. These pathogens are not only resistant to the carbapenems, but to many or most other classes of antibiotics that are effective and safe [11], such as broad-spectrum cephalosporins. Treatment of CRE with polymyxins and aminoglycosides is complicated by efficacy, pharmocokinetics, and toxicity [12]. Recently introduced treatment options such as meropenem-vaborbactam [13] or ceftazidime-avibactam are expensive [14], and clinical experience with their use is limited [13].

Although not all bacterial contact results in infection, patients can become asymptomatically colonized [15], leading to a higher probability of transmission to other patients [16], extended length of stay, increased carbapenem exposure, or infection, which increases mortality risk [17, 18] as high as 70% for patients in intensive care units [9]. As a result, the Centers for Disease Control has recommended active surveillance of patients at high risk [19]. Active surveillance, or screening without suspicion of infection, can identify asymptomatic carriers [20, 21] for subsequent intervention with infection control measures such as the use of contact precautions, decolonization, patient cohorting, or minimization of the use of invasive devices [15, 19], and may decrease rates of nosocomial transmission [22] and reduce morbidity, mortality, and hospitalization costs [23, 24].

Studies using active surveillance data typically use standard epidemiological methods such as case-control studies [25] or prospective observational studies [26] to determine a global statistic of infection or colonization and an assessment of risk factors and their importance. Because the infectious process is only partially observable and the data are highly dependent, mechanistic mathematical models that allow parameter estimation and hypothesis testing [27, 28] can be an important addition to the toolbox of epidemiologists, clinicians, and public health policy-makers. These methods can allow analysis of the dynamics of an outbreak and can use information about locations and times of colonization that are often obscured when data is aggregated for use with traditional methods. However, variants on the classic Kermack-McKendrick epidemic model [29]—which describes the spread of infectious disease in a population of uniformly-mixed, homogeneous individuals who may be disease susceptible, infective, or resistant (an SIR model)—are often poorly suited for describing pathogen spread within small populations with high patient turnover [30].

Stochastic models are naturally suited (and increasingly used [31]) to describe transmission and its variance amongst the small numbers of patients within hospital units. For example, several groups have used discrete-time [32] or continuous-time Markov chains to model and analyze hospital active surveillance data [32–40] for organisms such as methacillin-resistant *Staphylococcus aureus* (MRSA) [41, 42], vancomycin-resistant Enterococci (VRE) [38, 41, 43], carbapenem-resistant Enterobacteriae (CRE) [32], cephalosporin-resistant gram-negative rods (RGNR) [41], *Pneumococcus* [33], and/or *Pseudomonas* [38]. Earlier modeling efforts moved beyond simple Poisson or unstructured hidden Markov models, which assumed infections to be independent (appropriate for RGNR) [41], to incorporate two mechanisms of colonization: exogenous (patient cross-transmission) versus endogenous (arising from antibiotic pressure) for VRE [38, 41], *Pseudomonas* [38], MRSA [41], or CRE [32]. Later models incorporated other mechanisms such as healthcare workers as vectors [42, 44, 45], within-family versus community-acquired transmission [33], sporadic [37] or background colonization [35, 39, 40, 46], pre-existing colonization [32, 34, 37, 41, 47, 48], transmission from other rooms [46], or bacterial acquisition between hospital stays [34]. Notably, López-García & Kypraios created a generalized model of nosocomial spread that included multiple sources of transmission including patients, staff, and environment, and allowed calculation of the basic reproduction number [36].

Many models used point prevalence [34, 37, 44] from regular surveillance testing [38], but Cooper and Lipsitch used sparse microbiological and clinical culture results, although fitting parameters required multiple years of data [41]. Less frequently, groups directly tracked individual colonization statuses in the state of the model [32, 33]. Numerous groups subsequently applied Markov Chain Monte-Carlo (MCMC) methods to allow incorporation of prior knowledge about parameters [41, 49], estimation of sensitivity [34, 35, 39, 40, 47], and/or augmentation of data with individual patients’ possible but unobserved colonization times [34, 35, 39, 47]. Calculation of exact likelihood while incorporating partial observation data and unknown times of patient colonization was deemed an “intractable” problem by some groups [39, 47], although Bootsma et al. [32] estimates maximum likelihood in a discrete-time Markov model that uses test results to reduce the space of probable states and allows for varying occupancy of the unit.

In this work, we present a hybrid continuous-time/discrete-time Markov susceptible- infective-susceptible (SIS) model that allows direct calculation of likelihood while incorporating exact exit/entry times and incomplete observation data. This model tracks the discrete state of the unit, a binary string representing the colonization status of each patient within the unit. The state evolves continuously between events (tests or patient turnover) with possible patient transmission, but it can change instantaneously and discretely at the time of patient turnover with the entry of a new, potentially colonized patient and possible prior-to-new patient colonization event via a contaminated bed or surroundings. From a mechanistic standpoint, we describe a model of bacterial transmission and colonization tailored to analyze incomplete active surveillance data for carbapenem-resistant Enterobacteriaceae (CRE). Our mathematical model examines three mechanisms of bacterial colonization: **patient–patient transmission** (via healthcare providers or other vectors) [15], **prior-to-new patient transmission** (also referred to as patient-bed-patient transmission because it may occur via contaminated bed linens [26] or via the immediate surroundings [50]), and **pre-existing colonization** (in which patients have been colonized prior to hospital unit entry by exposure during previous hospitalizations or stays in long-term care facilities) [51, 52]. We will discuss 1) formulation of a hybrid discrete-time/continuous-time Markov model analogous to a SIS model (in which beds can be in either a colonized/uncolonized state), 2) event-driven simulation using data for patient exit and entry, 3) an inference method designed for incomplete active surveillance data that calculates parameter likelihood given all possible sequences of states consistent with (partial) observations, 4) methods to speed calculation of the likelihood function, 5) comparison with a reduced-state model similar to previously published SIS models [32, 34, 36], 6) results from maximum-likelihood parameter estimation, and 7) the contributions of this work in the context of past modeling and parameter inference efforts.

From an epidemiological standpoint, the model and methods presented in this paper can be used to improve estimation of patient–patient transmission from sparse data by using the individual time, location, and test result information for each patient, allowing parameter estimation from individual rooms and small hospital units. Furthermore, the incorporation of multiple routes of transmission allows breakdown of the overall colonization prevalence (total colonized patient-days) into the components attributable to mechanisms in excess of pre-existing colonization. From a technical standpoint, the methods for inference and parameter estimation allow exact determination of maximum likelihood parameters without requiring likelihood function estimation or sampling the distribution of possible realizations using MCMC techniques, even when the model has a large number of possible states. This inference method is generally applicable to discrete-state, discrete- or continuous-time Markov models applied to partial observation data. Although calculation of the likelihood function for any given set of parameters is expensive because it incorporates a matrix exponential [53], use of the full detailed state tracking individual patient colonization statuses and the exact entry/exit times enabled by the continuous-time model allows better parameter estimation compared to use of a reduced state model that tracks the number of colonized patients. We outline a number of mathematical and computational methods to make this calculation both feasible and practical. (Details of many of these methods are available in the appendices following the main body of the paper).

## Materials and methods

### Ethics statement

The Institutional Review Board of the NYU School of Medicine approved this study (IRB #08 818) on the “Epidemiology of KPC producing Enterobacteriaceae in New York City” with a waiver of consent: “Active surveillance for multi-drug resistant bacteria is a routine, well-accepted method for enhancing infection control in areas of high risk in the hospital setting. Only routine clinical specimens obtained for the active surveillance of KPC-E are used in this study.” The data were analyzed anonymously.

#### Active surveillance data

We used de-identified hospital surveillance and census data (available in S1 Data) for CRE colonization within a rehabilitation unit in a New York City academic center, a subset of the data used for a CRE case-control study by Swaminathan et al. [25]. The surveillance data consisted of perirectal swabs taken shortly after patient entry into the hospital unit and approximately weekly thereafter until exit. All events were drawn from an electronic medical record which placed timestamps on tests and entry/exit events to at least the date, hour, and minute. The swabs were cultured for CRE (primarily *Klebsiella pneumoniae*). Also available were clinical microbiological test results from cultures performed upon suspicion of infection. These data were combined with census data consisting of patient bed locations and turnover times for the study duration of 417 days. (The average patient length of stay was 14.1 days). We limited our analysis to 13 of 25 beds in the rehabilitation unit that were consistently used, as the remainder were either temporary or rarely used beds that stayed empty for the majority of the study duration. Additional information is available in S2 Appendix.

## Models

### Hybrid discrete-time/continuous-time Markov model

Consider a hospital unit of *n* beds filled with patients, all of whom are initially uncolonized with pathogen. We model the process of colonization and transmission as a hybrid discrete-time/continuous-time, discrete-state Markov chain [54, 55] in which a discrete-time Markov process is used to describe events occurring at patient turnover, but a continuous-time Markov chain is used to describe events occurring during time intervals in which no patients enter or leave.

The **detailed state** of the unit at time *t* is represented as a binary vector (or bit string) **b**(*t*). Each component *b*_*k*_ ∈ {0, 1} corresponds to the status of the patient in bed *k* at time *t*, for all *k* ∈ {1, 2, …, *n*}. Patients are considered uncolonized (*b*_*k*_ = 0) if they do not harbor pathogen, but colonized (*b*_*k*_ = 1) if they do, even if asymptomatic. These states are equivalent to the susceptible and infective states of an SIS model, which is a two-state variant of the classic three state susceptible-infective-resistant (SIR) model [29]. We assume beds are never empty, so there are only two possible states for each bed and only 2^n^ possible states of the unit. The **reduced state** of the unit is an integer representing the total number of colonized patients in the unit at a given time. In the detailed model, we choose to track the status of individual patients and not just the *number* of colonized patients, even though the detailed state determines the reduced state—but not conversely—simply by counting the number of colonized patients.

We assume three mechanisms in the model by which individual patients become colonized: **pre-existing colonization**, **prior-to-new patient colonization**, and **patient–patient transmission**. Here, pre-existing colonization is defined as carriage of pathogen upon entry into the unit. Prior-to-new patient colonization is transmission of pathogen from the prior bed occupant to the new patient immediately following, which can occur via contamination of the bed [26] or immediate surroundings [50]. Finally, patient–patient transmission can occur between any two patients who are simultaneously present in the unit via mechanisms such as the use of shared equipment or contact with health-care providers [15, 56]. Of the three processes, note that two occur (or can be thought of as occurring) instantaneously *during* patient turnover (pre-existing and prior-to-new patient colonization), whereas the third (patient–patient transmission) is considered to occur at any time *between* patient turnover events.

**Fig 1:**
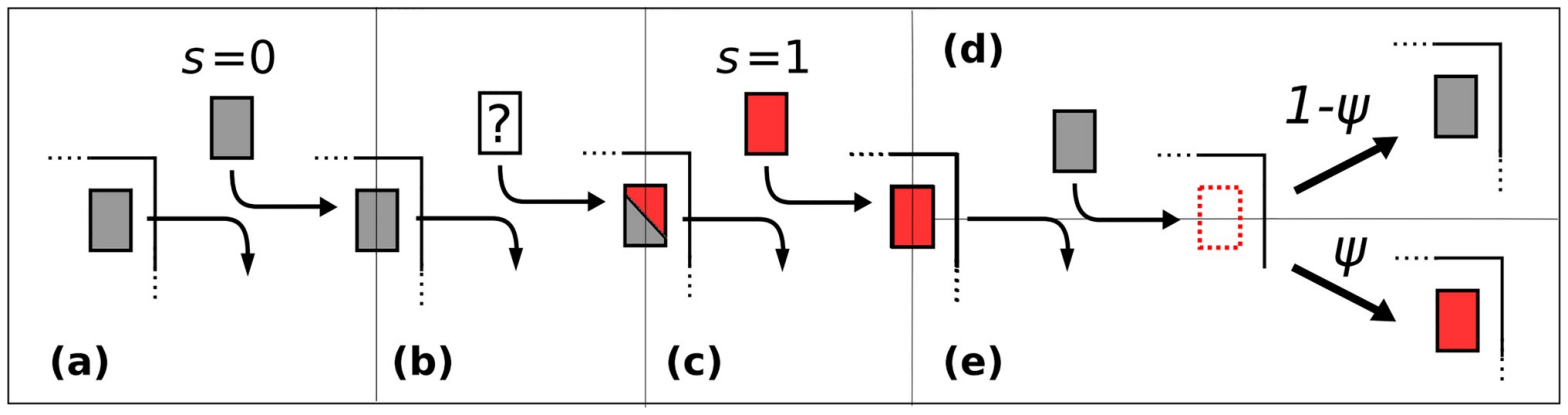
Possible exit/entry scenarios. Possible exit/entry scenarios, with *s* as an indicator variable for patient colonization (if known), *ϕ* as the probability of pre-existing colonization (if the new patient’s status is unknown), and *ψ* as the probability of prior-to-new patient colonization given that the prior patient was colonized: (a) replacement of an uncolonized patient by an uncolonized patient (*s* = 0 if status known); (b) replacement of an uncolonized patient by a patient of unknown status (colonized with probability *ϕ*); (c) replacement of a patient of any status with a colonized patient (*s* = 1 if status known); and replacement of a colonized patient by an uncolonized patient (*s* = 0 if status known, otherwise uncolonized with probability 1 − *ϕ*), who (d) remains uncolonized with probability 1 − *ψ* or (e) becomes colonized via prior-to-new patient colonization (probability *ψ*).

During exit/entry events, colonization can occur via prior-to-new patient colonization. Let *S* be a dichotomous random variable denoting the unknown colonization state of an entering patient, and let *ϕ* be the probability of **pre-existing colonization** (Fig 1b). *S* = 0 codes for uncolonized; *S* = 1 codes for colonized; Pr{*S* = 1} = *ϕ*; and Pr{*S* = 0} = 1 − *ϕ*. When the colonization state is known (not random), it is denoted by *s*, where (similarly) *s* = 0 for uncolonized (Fig 1a), and *s* = 1 for colonized (Fig 1c). If the new patient is uncolonized (*s* = 0 if status known, otherwise *S* = 0 with probability 1 − *ϕ*) and enters a bed in which the previous patient was colonized (Fig 1e), there is a probability *ψ* of **prior-to-new patient colonization**, which may occur via contamination of the bed or immediate surroundings. Alternately, the patient may remain uncolonized with probability (1 − *ψ*). We assume that exit, entry, and possible prior-to-new patient colonization occurs instantaneously during an **exit/entry event**.

**Fig 2:**
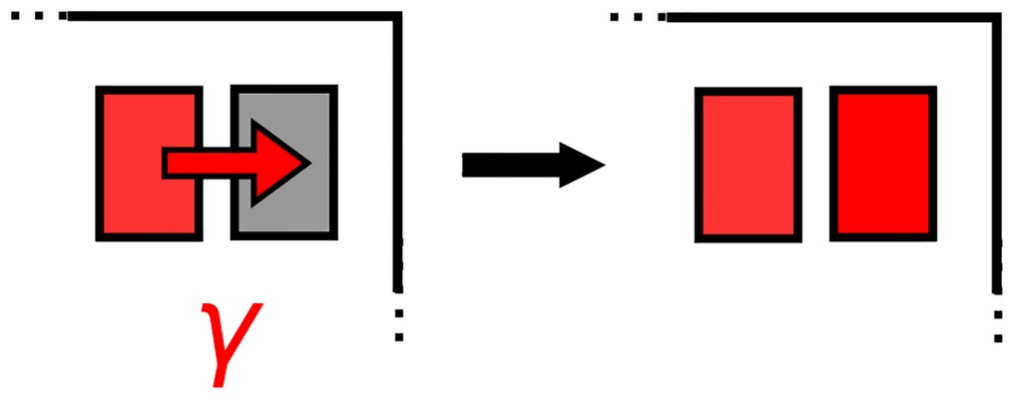
Transmission from colonized to uncolonized patient. Transmission from colonized to uncolonized patient at rate *γ* per day per colonized-uncolonized patient pair.

Between exit/entry events, **patient–patient transmission** can occur from colonized to uncolonized patients. For any given uncolonized patient, the fixed probability per unit time of a colonization event per colonized patient in the hospital unit is *γ* (Fig 2). Thus, we are assuming that the overall patient–patient transmission rate in the hospital unit is linearly proportional to the number of *pairs* of colonized and uncolonized patients present at a given time. Although continuous-time transmission is “restarted” at each exit/entry event, the model is a “memoryless” Markov model [55] that depends only on the present state to determine the next state without regard to history. At the discontinuity of an exit/entry event, the number of colonized patients may instantaneously increase or decrease by 1, which then would change the unit transmission rate (*iγ*, where *i* is the number of colonized patients present).

Of note, we use the term “rate” (the expected number of events per unit time) interchangeably with the term “probability per unit time,” but distinguish it from “rate constant,” which is a reaction rate coefficient in chemical kinetics that is *not* equivalent to the probability per unit time [57, 58]. We use “probability per unit time” as a shorthand for *instantaneous* probability per unit time, defined as the probability per unit time as the time interval decreases to an infinitesimal size (equivalent to a hazard function from survival analysis [59]):

$$\begin{array}{c} \lim_{\Delta t\to 0}\frac{P(\text{event}\;\text{occurs}\;\text{in}\;\text{the}\;\text{time}\;\text{interval}\;(t,t+\Delta t))}{\Delta t} \end{array}$$

We define *γ* as the instantaneous probability per unit time of a patient-patient transmission event occurring from the initial time at which patient-patient transmission can occur (here assumed to be *t*_0_ = 0) to time *t*. The probability of no event occurring during the time interval (0, *t*) is *e*^−*γt*^ (equivalent to a cumulative survival function [59]), so the probability of at least 1 event occurring during the time interval is 1 − *e*^−*γt*^.

#### Changes in the state of hospital units

**Fig 3:**
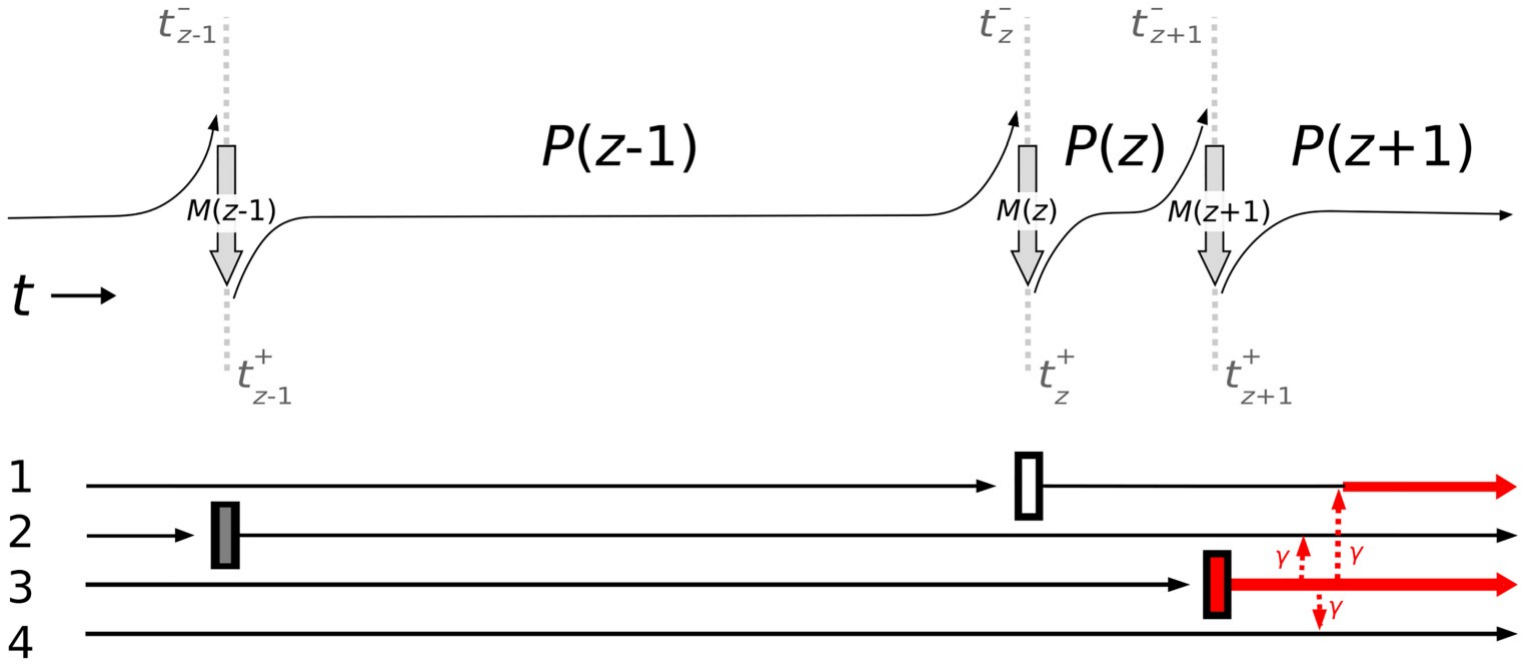
Overview of all processes of colonization and transmission. Overview of all processes of colonization and transmission. The top diagram shows the two types of transition matrices. *M*(*z*) is the exit/entry probability matrix, which describes probabilities of various outcomes upon patient turnover and possible prior-to-new patient transmission at an exit/entry event. *P*(*z* − 1) is the non-turnover transition *probability* matrix, which is derived from the patient–patient transmission *rate* matrix and describes what happens from one turnover event to the next. The bottom diagram shows the statuses of individual beds within an example four-bed hospital unit. The vertical bars represent a patient turnover event. The vertical bar color represents the status of the incoming patient: white if uncolonized, red if colonized, and gray if unknown. The horizontal lines depict the colonization status of the patient in a particular bed: a thin black line means the patient is uncolonized, but the thick red line means the patient is colonized. The dashed red arrows represent the probability per unit time of patient–patient transmission between pairs of colonized-uncolonized patients.

In the next section, we describe how probabilities evolve for the change in state of the entire hospital unit at and between events as a result of changes in the colonization status of individual patients. Let *T* be a vector of arbitrary length of the known, arbitrary times at which patient turnover occurs. We assume that patient replacement is instantaneous and the status of the *z*-th incoming patient may be known or unknown. A patient that leaves at time $t_{z}^{-}$ is immediately replaced a new patient at time $t_{z}^{+}$ (Fig 3). Here, $t_{z}^{-}$ is the limit approaching *t*_*z*_ from the left and $t_{z}^{+}$ is the limit approaching *t*_*z*_ from the right, given that time is pictured as flowing from left to right as in Fig 3.

Let **p**(*t*) be the probability distribution at time *t* of the different possible states of the unit. That is, **p**(*t*) is a vector with 2^n^ entries, each of which refers to a state of the *n*-bed unit as a whole and gives the probability that the hospital unit is in the corresponding state. Notice that in our model, pre-existing and prior-to-new patient colonization occur only during patient exit/entry events, but patient–patient transmission occurs solely *between* exit/entry events during **non-turnover intervals**. The probability distribution changes in a discrete manner at an exit/entry event, which begins at the time of the prior patient’s exit ($t_{z}^{-}$) and ends after the new patient’s entry and possible prior-to-new patient colonization ($t_{z}^{+}$). Between exit/entry events, no patient turnover occurs, and the probability distribution evolves continuously (Fig 3).

Transition probabilities for changes in the state of the hospital unit that occur *during* the *z*-th exit/entry event are represented as entries within **a discrete-time Markov transition** ***probability*** **matrix** *M*(*z*) (described in S1F Appendix). In contrast, the transition *rates* for state changes that occur *between* patient exit/entry events are represented as entries within the **continuous-time transition** ***rate*** **matrix *R*** (described in S1G Appendix). The cumulative effect of these changes can be written [60, 61] as a *probability* matrix *P*(*z*) that examines the change in the state of the hospital unit from the beginning to the end of a discrete time interval, as shown in S1H Appendix.

### Simulation

Given input parameters *γ*, *ϕ*, and *ψ*; a list of *Z* − 1 exit/entry events at arbitrary times *T* = {*t*_1_, …, *t*_*Z*−1_} that occur in beds *K* = {*k*_1_, *k*_2_, …, *k*_*Z*_}; and, optionally, a list of arbitrary states *S*(*z*) of the entering patients at time *t*_*z*_, we can create a simulation of the hospital unit for the period *t*_0_ to *t*_*Z*_. (Here, we assume that at time *t*_0_, the hospital unit is completely uncolonized, and the end of the simulation period occurs after the time of the last exit/entry event at time *t*_*Z*−1_. The simulation can also be performed with other initial states if desired).

Simulation of the hybrid continuous-time/discrete-time model requires three steps: 1) choosing a list of **arbitrary exit/entry times** from data or simulation; 2) **simulating** the **exit/entry events** of pre-existing colonization and possible prior-to-new patient colonization using the probabilities *ϕ* and *ψ*, and 3) using **event-driven simulation** (also known as the Gillespie Method [57, 62, 63]) and the parameter *γ* to simulate patient–patient transmission *between* exit/entry events.

First, we used the exact exit/entry times from the rehabilitation unit census data. However, it is also possible to create simulations using turnover times from other data sets or other methods, such as generating these times using the assumption that exit/entry is a Poisson process governed by the turnover time (the inverse of the mean length of stay [64]).

Second, we assign patients an initial colonization status upon entry into the unit and a final status after contact with the bed. If the patient’s colonization status was previously known to be uncolonized or colonized, the initial colonization status was tentatively set to be 0 or 1, respectively. Incoming patients whose colonization status was unknown were given an initial status of 0 with probability 1 − *ϕ* or status of 1 with probability *ϕ*. Then, uncolonized new patients (initial status 0) entering a bed previously occupied by a colonized patient were given a final colonization status of 0 or 1 with probability 1 − *ψ* or probability *ψ*, respectively. Patients who entered initially colonized remained colonized after contact with the bed, regardless of the previous patient’s colonization status, and were given a final status of 1. Details about exit/entry event simulation are found in S1B Appendix.

Last, we performed stochastic simulations of patient–patient transmission using **event-driven simulation** [57, 62, 63]. Recall that the probabilities per unit time of patient–patient transmission are represented as a continuous-time Markov chain for the interval between two exit/entry events at times *t*_*z*−1_ and *t*_*z*_ in which no patient turnover occurs. During this interval, the only possible event is patient–patient transmission, which occurs at rate *γ*. Event-driven simulation can be used to determine the time and location of patient–patient transmission events using a variant [63] on the Gillespie method [57, 62]. This method uses transition rates from a current state to multiple possible final states to create a realization of the final state of the system after transition. Because this simulation method uses random numbers as input, individual realizations will be different, but in aggregate, their statistics will approach that of the original transition rates. Details for simulating patient–patient transmission between exit/entry events are described in S1C Appendix.

### Inference method

**Fig 4:**
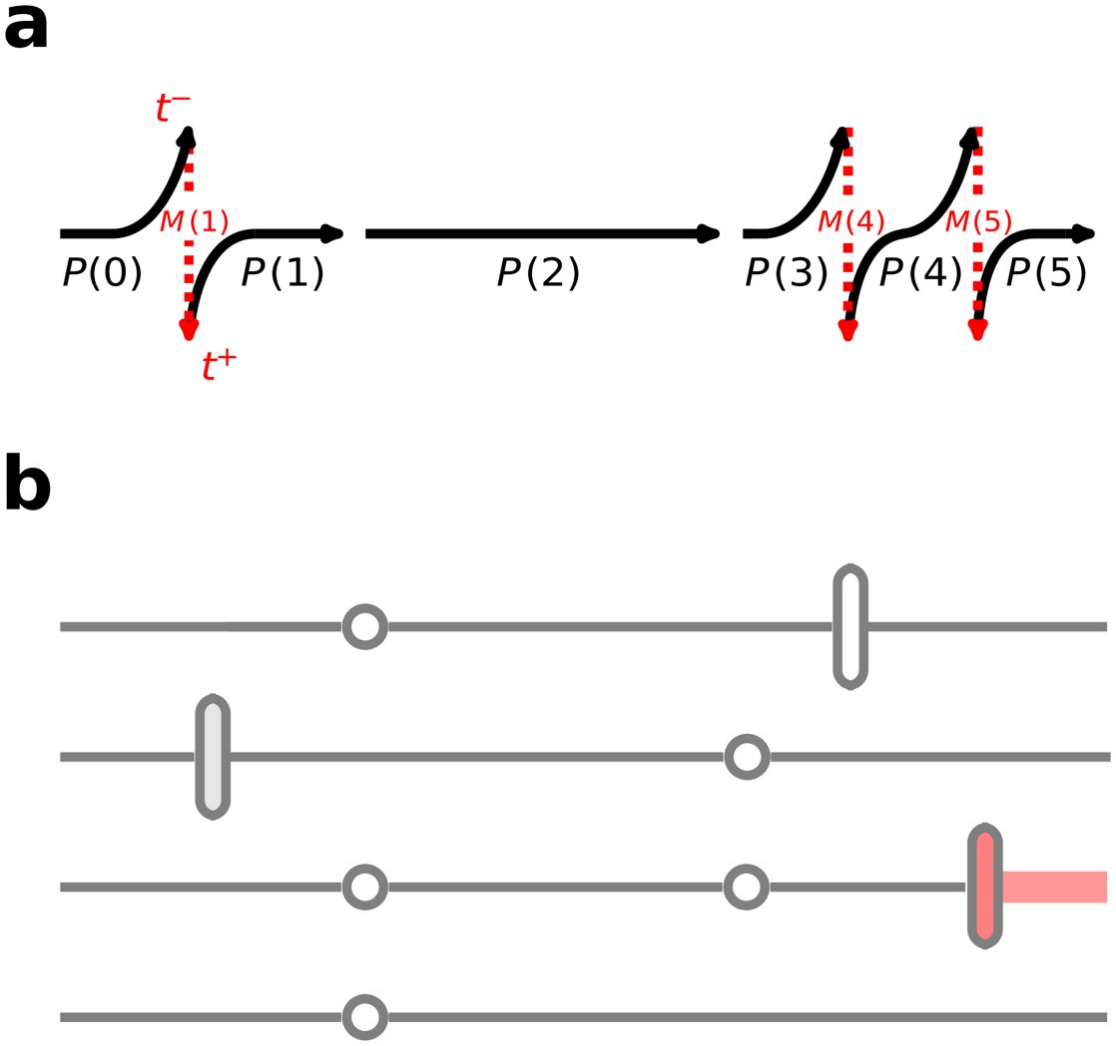
Example event timeline for a surveillance study performed on a 4-bed unit (with time flowing left to right). (a) Surveillance study timeline divided into non-turnover intervals (black arrows) by tests (spaces between black arrows) and exit/entry events (dotted red arrows). Each non-turnover matrix for the interval *t*_*z*_ to *t*_*z*+1_ has a corresponding matrix *P*(*z*), and each exit/entry event at time *t*_*z*_ has a corresponding *M*(*z*) matrix. (b) The events of a surveillance study in which each horizontal line represents the history of a bed, which may include exit/entry events (vertical bars) or tests (open circles). The color of the vertical bar indicates whether the incoming patient was uncolonized (white), colonized (red), or untested with unknown status (gray).

The inference method in this paper is designed for use with the partial observations and arbitrary test and patient turnover times often found in active surveillance data. As shown in Fig 4, entering and exiting patients may have an unknown status (gray vertical bar) or be known to be uncolonized (white bar) or colonized (red bar). Patients are tested at arbitrary times (open circles), but typically not every patient in the hospital unit is tested at the designated time(s). The possible *true* state of the hospital unit can only be known if all patients are tested at the same time, but in practice, the state of the unit is often partially rather than fully observed. All test results obtained are assumed to be accurate.

In the next section, we describe cleaning and pre-processing of the active surveillance dataset, including **data augmentation**, a method for extrapolating test results forward and backward in time based on some simple assumptions (similar to the assumptions of Bootsma et. al [32]). We then describe a likelihood function, that is, the probability of a single sequence of observations given a set of arbitrary parameters. Finally, we describe an inference method that maximizes this likelihood function. Because direct calculation of the likelihood function is computationally expensive, we outline multiple techniques that can be used to streamline this calculation, including a method to “compress” a patient–patient transmission matrix *P*(*Z*) from size 2^n^ to *n* + 1 square (S1N Appendix); use of Jordan form to find the analytical solution to the matrix exponential of the rate matrix *R*(*t*) (S1O Appendix); and other computational techniques to reduce the required memory and time required for a single likelihood calculation. We used these methods in conjunction with MATLAB’s built-in optimization function **fmincon** to calculate the maximum-likelihood parameters for our data set.

#### Data pre-processing

Because the mathematical model assumes that no time elapses between exit and entry, we assumed that the “exit/entry” events of the model occurred at the recorded time of patient entry in the dataset. We assumed that once colonized, patients remained colonized throughout their stay, because even intentional attempts at decolonization have limited efficacy [65, 66]. Negative test results following positive test results were assumed to be false negatives, as intestinal excretion of CRE can be intermittent [67]. Patients who are uncolonized at one test time were assumed to be uncolonized at all times *prior* to the test, from and including the time of entry; patients found to be colonized at a particular test time were assumed to be colonized for all times at and *after* the test until exit. (The patient colonization status is still unknown after a negative test, before a positive test, and between a negative and a positive test because the exact time of conversion, if one occurred, is unknown). Thus, at each time at which testing was performed within the hospital unit, inferred test results were given to all other patients whose colonization statuses could be inferred. The final augmented data set of actual and inferred test results was used for all parameter estimation within this paper. Additional statistics and information on data pre-processing are available in S2 Appendix.

#### Probability of a sequence of states

Consider a sequence of states and their corresponding matrices (either exit/entry *M*(*z*) or patient–patient transmission probability *P*(*z*) matrices) that occur at given times *t*_*z*_. If all patients are tested during an observation, the state of the hospital unit will be completely known at that time. If not all patients are tested, only incomplete data regarding the state of the hospital unit will be available, as shown in the example testing scheme of Fig 4. For convenience, we will re-index the states **b**(*z*) and the corresponding matrices in sequential order with integer indices *j*, allowing us to rid our notation of limits (such as $t_{z}^{-}$ or $t_{z}^{+}$).

The re-indexed states and matrices will be notated as **a**(*j*) and *W*(*j*), where *W*(*j*) is a generic transition matrix that denotes either an exit/entry matrix *M*(*z*) or the patient–patient transmission matrix *P*(*z*) describing the probability of transitioning from state *j* − 1 to state *j*. Notice that *M*(*z*) describes a state transition occurring instantaneously at exit/entry/bed contact, but *P*(*z*) describes the probability of the state transition between two events at times *t*_*j*−1_ and *t*_*j*_. The *P*(*z*) probability matrix is created by exponentiating the continuous-time patient-patient transmission rate matrix over any interval of time between events such as exit/entry or tests. Thus, different *P*(*z*) matrices may represent probabilities of state transitions over time intervals of varying lengths, unlike a typical discrete-time Markov model which calculates the probability over fixed time intervals such as a day.

Here, *p*_0_ is the initial probability distribution, so *p*_0_(**a**) is the probability that **a**(0) = **a**, e.g., that if no time elapses, the state of the unit is the initial state. *W*_**a**(*i*)**a**(*j*)_(*i*) is the probability of transitioning from state **a**(*i*) at the *i*th event to state **a**(*j*) at the *j*th event. Notice that any amount of time can elapse between these events, so multiple instances of patient-patient transmission may occur between exit/entry and/or test events.

In the case of partial testing, there will be multiple possible states consistent with observations. For example, consider the first set of tests at time *t*_2_. If we assume all test results (for the white open circles shown) are negative, then the partially observed state of the unit is 00?0. The two possible underlying states consistent with test results are {0000, 0010}.

Let *B*(*j*) be the set of all possible states consistent with test results at event *j*. Each state vector **b**(*j*) in the set *B*(*j*) will have some components *b*_*k*_ (for *k* in the set of tested beds) whose status is known from test results, but the remaining components can take on any combination of values (0 or 1). Notice that if all tests are performed, the state of the system will be known and *B*(*j*) will have only one element. If no tests are performed, all states are possible, so *B*(*j*) will have 2^n^ elements. Thus, every observation with *m* tests performed (for *m* ∈ {0, 1, …, *n*}) yields a set of 2^*n*−*m*^ possible states consistent with its test results. The true state of the system *X*(*j*) at event *j* is contained within the set of possible states *B*(*j*). Therefore, the whole set of observations gives us the following information about the sequence of true system states at all of the test times:

$$\begin{array}{c} \left[X(0)\in B(0)]\;\text{and}\;[X(1)\in B(1)]\;\text{and}\;\ldots \;\text{and}\;[X(\zeta )\in B(\zeta )\right] \end{array}$$

The likelihood (a priori probability) P of this set of observations is

$$\begin{array}{c} P=\text{Pr}([X(0)\in B(0)]\;\text{and}\;[X(1)\in B(1)]\;\text{and}\;\ldots \;\text{and}\;[X(\zeta )\in B(\zeta )]) \end{array}$$

For brevity, we denote the sequence of states **i** = **a**(0), **j** = **a**(1), **k** = **a**(2), **y** = **a**(*ζ* − 1), and **z** = **a**(*ζ*). Then the likelihood equation can be written as

$$\begin{array}{c} P=\sum_{i\in B(0)}p_{0}\left(i\right)(\sum_{j\in B(1)}W{\left(0\right)}_{ij}(\sum_{k\in B(2)}W{\left(1\right)}_{jk}\cdots (\sum_{z\in B(\zeta )}W{\left(\zeta -1\right)}_{yz}))) \end{array}$$ (1)

(Note that this method is similar to Baum’s recursion method as used in the work of McBryde et al. [37]. The following method of obtaining submatrices is similar to the method by Bootsma et al. of reducing the number of possible states by incorporating observations using forward and backward vectors [32]).

Eq 1 can be written in matrix notation (and evaluated that way in MATLAB) if we introduce the following subvector and submatrices. Let *p*_0_(*B*(0)) be the row vector with entries of initial probabilities for each of the possible initial states in set *B*(0). Let *W*(*j*, *B*(*j*), *B*(*j* + 1)) be the rectangular submatrix of *W*(*j*) containing the rows corresponding with the states specified in *B*(*j*) and the columns corresponding with the states specified by *B*(*j* + 1). Finally, let *U*(*B*(*ζ*)) be a column vector with all elements equal to 1 and the same number of elements as contained in *B*(*ζ*). Then the overall probability is

$$\begin{array}{c} P=p_{0}(a(0))\;\;W(0,B(0),B(1))\;\;W(1,B(1),B(2))\;\ldots \;W(\zeta -1,B(\zeta -1),B(\zeta ))\;U(B(\zeta )) \end{array}$$ (2)

To find the parameters for the prior-to-new patient probability *ψ* and the transmission rate *γ*, we must maximize the likelihood P with respect to a particular sequence of observations: patient exit/entry events, the corresponding new-patient colonization statuses, and the test results of particular beds at given times.

#### Finding maximum likelihood parameters and error ranges

**Fig 5:**
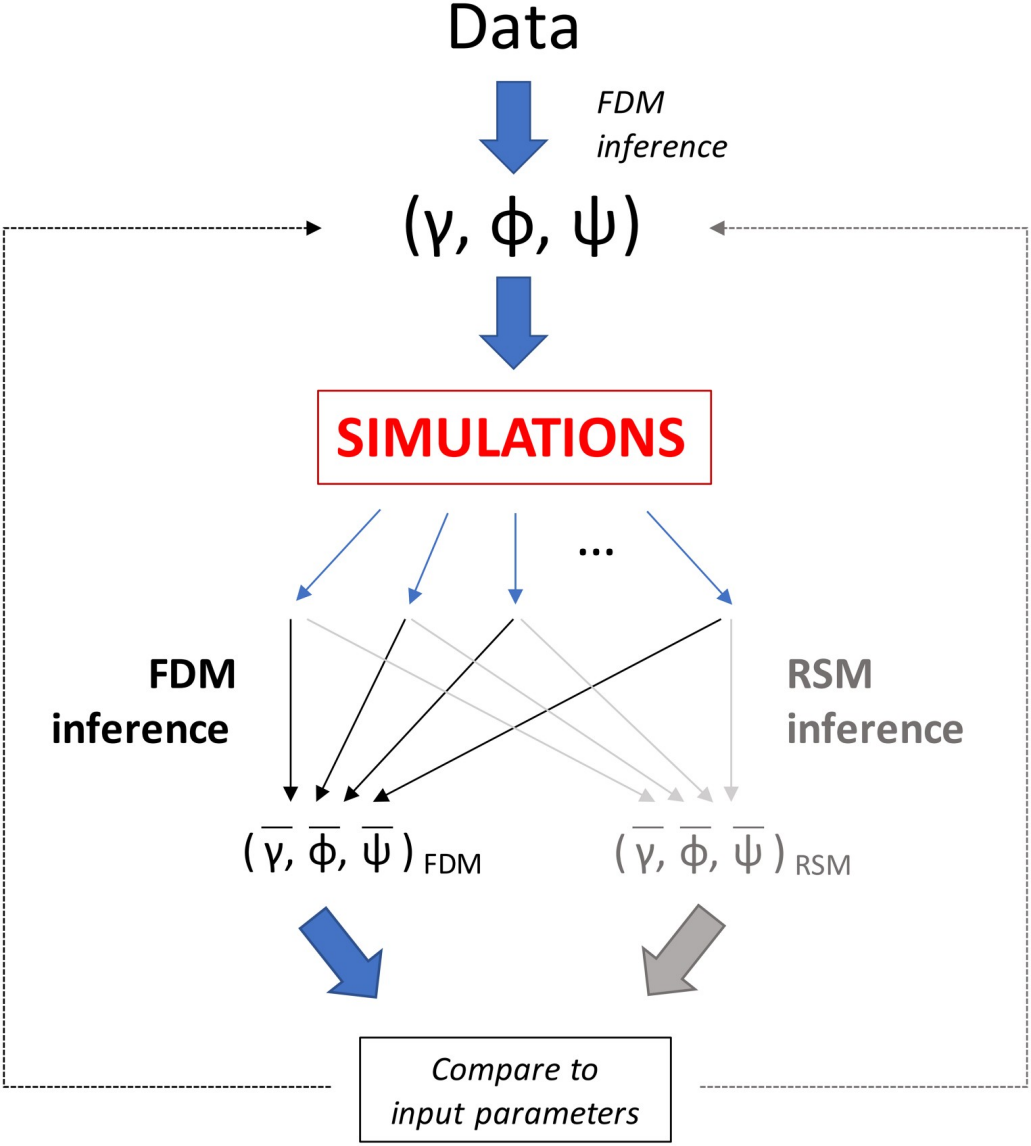
Comparison of inference methods of the full detailed model (FDM) versus the reduced state model (RSM) using simulations generated from the same set of input parameters (*γ*, *ϕ*, *ψ*). First, the dataset from the rehabilitation unit was used to find the best-fit parameters using the FDM. These parameters were used to generate simulations. The FDM and RSM inference methods were used to generate two sets of best-fit output parameters. The mean best-fit output parameters (*γ*, *ϕ*, *ψ*)_*FDM*_ and (*γ*, *ϕ*, *ψ*)_*RSM*_ from the FDM and RSM inference methods, respectively, were found and then compared to the input parameters (*γ*, *ϕ*, *ψ*).

We used the function P(B;γ,ϕ,ψ) (Eq 1) as the objective function to determine the likelihood of a sequence of observations *B* given input parameters *γ*, *ϕ*, *ψ*. The observations consisted of test times, locations, and results from active surveillance data for an *n*-bed hospital unit as well as census information (exit/entry times, the corresponding beds in which turnover occurred, and the colonization statuses of incoming patients if available). We determined the best-fit parameters by finding the maximum of the log-likelihood (the logarithm of Eq 1) using the MATLAB function **fmincon** with the following constraints: *ϕ* and *ψ* were probabilities with values between 0 and 1. (The parameters themselves remained on a linear scale). The patient-patient transmission rate *γ* is a probability per unit time that can have any non-negative value depending on the units chosen. Because estimates from epidemiological studies [68] or mathematical models [35, 39] suggested that the overall rate of transmission was less than than 25 cases per 1000 patient-days of exposure (0.025), we chose a generous parameter search range between 0 and 2. An initial search of the space suggested a single maximum lay between 0 and 0.5, so the final parameter search was restricted to values between 0 and 0.5. Example plots from the likelihood landscape for *γ* ∈ [0, 1] are shown in S1I Appendix. Error ranges were computed as described in Fig 5.

#### Techniques to improve inference

The continuous-time patient-patient transmission rate matrix allows use of exit/entry and test events at arbitrary times. However, this flexibility comes at a price: matrix exponentiation must be performed for each of the time intervals between events (approximately 500 intervals of different lengths for the rehabilitation unit data).

Calculating a matrix exponential is an inherently difficult problem because confluent or nearly confluent eigenvalues leads to a loss of accuracy, but algorithms that avoid eigenvalues tend to require more computer time and may be adversely affected by roundoff error [69, 70]. Finding the matrix exponential of the full detailed patient-patient transmission rate matrix becomes computationally expensive as the number of beds (*n*) increases because the number of matrix entries increases exponentially (as 2^2*n*^). For units with a small number of beds (for example, *n* = 4 beds), the time needed is trivial because the matrices are 16 by 16, but for an *n* = 13 bed room, the full detailed state rate matrix has a size of 8192 by 8192. Consequently, if it is possible to decrease the dimensions of the transmission rate matrix or to factor the rate matrix so the matrix exponential can be calculated using only scalar exponentials, this step can be sped up significantly.

As described in S1N Appendix, we “compress” the patient-patient transmission rate matrix from size 2^n^ by 2^n^ to size *n* + 1 by *n* + 1 by converting entries in the full detailed matrix to their reduced matrix equivalents, similar to previous work analyzing SIS epidemic dynamics on a network of fully “connected” individuals [71]. Matrix exponentiation can then be performed on the smaller matrix and the entries converted and “exploded” back to their correct locations in the full detailed matrix. This is a tremendous advantage because the number of reduced states increases only linearly with the number of beds (as *n* + 1) [71]. Although we cannot diagonalize the matrix because it is defective, we were able to find the analytic solution for the Jordan matrix exponential, as shown in S1O Appendix. This solution allows us to calculate scalar exponentials of the Jordan matrix to find the matrix exponential.

#### Implementing the inference method

The surveillance data, microbiological test results, and hospital census data were pre-processed using Python and MATLAB (R2015b). The simulation and inference methods were implemented in MATLAB. Parameter estimation and simulation were performed in parallel using a combination of MATLAB and Bash scripts on Phoenix (now BigPurple), the high-performance computing cluster at New York University Medical Center. Parallel processing was used for initial searches of parameter space and parallel simulations of the forward model.

### Reduced state model

#### Reduced state model

The reduced state model is a continuous-time SIS Markov model in which the patient turnover is approximated as a Poisson process [72] in which patient exit/entry events occur continuously and independently at a constant average rate *β*. In essence, the reduced state model is a continuous-time classic birth and death process [73] applied to a susceptible-infective-susceptible epidemic model. The time between these exit/entry events is described by the exponential distribution, a memoryless probability distribution in which 1/*β* describes the mean time between exit/entry events (e.g., the average length of stay) [64]. Additional information and data supporting this assumption are described in **Fig B in** S2 Appendix.

The rate of increase (*f*_*i*_) in the number of colonized patients from *i* to *i* + 1 is *f*_*i*_ = *γi*(*n* − *i*) + *β*(*n* − *i*)*ϕ*. Notice that *f*_*i*_ is governed by parameters for the turnover rate *β*, the patient–patient transmission rate *γ*, and the probability of pre-existing colonization *ϕ*. However, the rate of decrease is governed only by the parameters *ϕ* and *ψ* (the prior-to-new patient colonization probability): *g*_*i*_ = *βi*(1 − *ϕ*)(1 − *ψ*).

Let *P*_*i*_ be the probability of being in state *i*. As time passes, the probability of being in a particular state will change. For the state with *i* patients colonized, the differential equation for the change in *P*_*i*_ is *dP*_*i*_/*dt* = *f*_*i*−1_ *P*_*i*−1_ + *g*_*i*+1_ *P*_*i*+1_ − *f*_*i*_ *P*_*i*_ − *g*_*i*_ *P*_*i*_. At steady state, there is no change in the probability of being in any particular state, so the time derivatives of *P*_*i*_ can be set equal to zero. If we solve *dP*_0_/*dt* = *g*_1_ *P*_1_−*f*_0_ *P*_0_ = 0 for *P*_1_, we find that *P*_1_ = *f*_0_ *P*_0_/*g*_1_. By induction, we find the equations for *i* ∈ {2, …, *n*} are *P*_*i*_ = *f*_*i*−1_ *P*_*i*−1_/*g*_*i*_. This equation shows the probability for the next higher state *P*_*i*+1_ in terms of the present state *P*_*i*_. Substituting in recursively and solving for the probability *P*_*i*_ in terms of *P*_0_, for *i* ∈ {1, 2, …, *n*}, we find

$$\begin{array}{c} P_{i}=\prod_{j=0}^{i-1}\frac{f_{j}}{g_{j+1}}P_{0} \end{array}$$ (3)

Because the sum of probabilities over all possible states is 1, we can solve for *P*_0_:

$$\begin{array}{c} P_{0}=\frac{1}{1+\sum_{i=1}^{n}(\prod_{j=0}^{i-1}\frac{f_{j}}{g_{j+1}})} \end{array}$$ (4)

The equations for *P*_*i*_ and *P*_0_ are general and hold regardless of the actual rates *f*_*j*_ or *g*_*j*_. A detailed description of the reduced model is found in S1K Appendix.

The probability of all possible sequences of states consistent with (possibly incomplete) observations is analogous to the likelihood equation of the full detailed model (Eq 2, but the matrices have dimensions of *n* + 1 by *n* + 1, not 2^n^ by 2^n^. In the reduced model inference method (described in S1K Appendix), we fit the probability distribution of states for the theoretical values against the distribution of states from actual data to estimate the minimum-error parameters.

#### Total prevalence

Total prevalence (*TP*), also known as the colonization pressure, can be defined as the fraction of patient-days during which patients are colonized. The theoretical total prevalence can be calculated from the reduced state model for any given set of parameters.

The total prevalence is the sum of the probabilities of each state weighted by the number of colonized patients in each state: *TP* = 0*P*_0_ + 1*P*_1_ + 2*P*_2_ + … + *nP*_*n*_. In terms of the rates of increase (*f*_*i*_) or decrease (*g*_*i*_), the total prevalence can be written as

$$\begin{array}{c} TP=\sum_{i=0}^{n}iP_{i}=\sum_{i=0}^{n}(\prod_{j=0}^{i-1}\frac{f_{j}}{g_{j+1}})iP_{0} \end{array}$$ (5)

Total prevalence, or the probability that at least 1 patient is colonized, is also equivalent to 1−*P*_0_ (with *P*_0_ defined in Eq 4). We can estimate the theoretical contribution of each colonization mechanism to total prevalence by varying the input parameters *γ*, *ϕ*, and *ψ* that govern the rates of increase *f*_*i*_ and decrease *g*_*i*_ in the number of colonized patients.

### Comparison of reduced and full detailed model results

Fig 5 shows the process used to estimate error and check performance of our model. First, we used the best-fit parameters from the actual dataset (Table 1) to generate synthetic data. For this, we used the identical scheme of exit/entry events and testing times/locations as the true data from the rehabilitation unit. Then, we applied the inference method for the full detailed model to the simulated data sets to find the estimated parameters, their mean recovered values, and standard deviations for *γ*, *ϕ*, and *ψ*. Finally, for each parameter, we found the mean value over the 1265 trials and used the standard deviation to compute an error range (Table 1).

**Table 1:** Comparison of input parameters for simulations versus the mean estimated value (MEV) and standard deviations (SD) of parameters recovered using the inference method from the full detailed state model (FDM) or the reduced state model (RSM).

	Input	FDM MEV ± SD	FDM error	RSM MEV ± SD	RSM error
*γ* (per day)	0.002029	0.001869 ± 0.000834	0.0788	0.002310 ± 0.000417	0.1386
*ϕ*	0.049700	0.048655 ± 0.012559	0.0210	0.066839 ± 0.015914	0.3448
*ψ*	0.000946	0.022321 ± 0.036288	22.6023	0.353808 ± 0.004715	373.119
SSE		0.000458		0.124806	

Here, SD = standard deviation, SSE = sum of squares error. The model error per parameter is simply the fraction error |*x* − *x*′|/*x*, for true value *x* versus estimated value *x*′.

## Results

We applied the full detailed state model (FDM) inference method to the 13-bed rehabilitation unit to determine maximum-likelihood parameters. We estimated the maximum-likelihood parameters for the 13-bed rehabilitation unit to be *γ* = 0.00203/day per colonized/uncolonized patient pair, *ϕ* = 0.0497, and *ψ* = 0.000946. For a scenario in a 13-bed unit in which 1 patient is colonized, there are 12 colonized-uncolonized patient pairs, so the probability per day of a transmission from the colonized patient to *any* uncolonized patient in the entire unit is about 0.02. A pre-existing colonization probability of about 5% suggests that about 5 out of every 100 patients is colonized. Finally, having a very small best-fit parameter for bed-patient colonization suggests that the probability of transmitting bacteria from a colonized patient to the bed or environment and subsequently to the next patient entering the bed (if uncolonized) essentially does not occur or is not detectable using the FDM method with available data.

**Fig 6:**
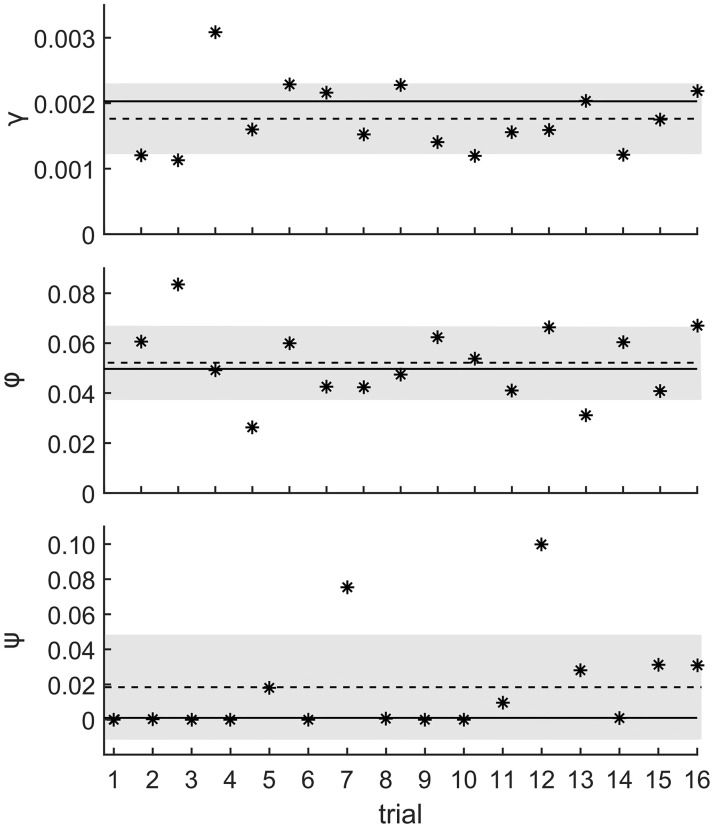
Range of parameter estimates inferred from 1265 simulations of the full detailed model. Range of parameter estimates inferred from 1265 simulations of the full detailed model created using the input parameters *γ* = 0.00203 per colonized/uncolonized patient pair per day, *ϕ* = 0.0497, and *ψ* = 0.000946. Each star represents the estimated maximum-likelihood parameter for an individual trial, a dashed line represents the mean best-fit parameter over the 1265 trials, and the gray region shows the standard deviation from the mean best-fit parameter. The bold line shows the input parameter value.

In order to evaluate the reliability and reproducibility of these estimates, we used the best-fit parameters, exit/entry times, and testing schema from the actual hospital data as starting points for simulations. “Observations” (using actual hospital test times and locations) were performed on the simulation data, and the inference methods using the full detailed model or reduced state model were applied to the simulated data to find best-fit parameters. The inferred parameters were then compared with the input parameters for the simulations to evaluate how well each inference method worked. A summary table of parameters, error ranges, and the sum of squares error for the full detailed model and the reduced model are shown in Table 1. Visualizations of best-fit parameter results for individual simulations are available in Fig 6 for the FDM and **Fig G-Fig I in** S1L Appendix for the RSM. Fig 6 shows the results of parameter estimation using the full detailed state model (FDM) inference method applied to 1265 simulations that were created using the above maximum-likelihood parameters as input. The mean recovered parameters were *γ* = 0.0018±0.0008 per day per colonized/uncolonized patient pair, *ϕ* = 0.05±0.01, and *ψ* = 0.02±0.04, where the error range is the standard deviation of estimated parameters. Applying the reduced state model (RSM) inference method on 1265 simulations (**Fig G-Fig I in** S1L Appendix), we found the average estimated best-fit parameters to be *γ* = 0.0023±0.0004 per day for each colonized/uncolonized patient pair, *ϕ* = 0.07±0.02, and *ψ* = 0.35±0.005. Note that for the FDM, the “true” (input) parameters are all contained within the error range. The RSM also has relatively good estimates for the input *γ* and *ϕ*, both of which are contained within the error range. However, the RSM estimate for *ψ* is highly—although consistently—inaccurate (with a narrow error range); this suggests that the RSM model is not capable of accurately inferring *ψ*. As described at length in the discussion regarding the **non-equivalence of the full and detailed states for inference**, patient-patient transmission for *γ* involves only the *number* of simultaneously colonized patients in the unit, which is captured in the state data for both the FDM and RSM. However, determination of whether colonization of a new patient from a prior patient has occurred requires knowing *which* patients in the unit were colonized and when, not just how many—information that is captured by the FDM but not RSM.

For the full detailed model, the error in the estimate for *ψ* is high and remains so despite the large of number of simulations performed, each of which was created with the exact exit/entry events, observations, and length of time as the actual data from the study. These simulations are statistically identical and have a finite amount of data. Because the error involved in parameter fitting has components of both bias and statistical error, the large number of trials performed will only eliminate the statistical error, not the bias. In the case of *ψ*, the parameters are constrained to be non-negative, introducing a bias, especially as the value for *ψ* is effectively zero. These results reveal that the mechanism of prior-to-new patient colonization is relatively unimportant compared to pre-existing colonization and patient-patient transmission. This suggests that for a realistic hospital surveillance situation with moderate testing compliance and low frequency of prior-to-new patient colonization, estimating the *value* of *ψ* will be difficult using the FDM method. At larger values of *ψ*, however, the FDM method may be able to produce better quantitative estimates.

### Effect of a long-stay patient

In the actual rehabilitation unit data, a single patient was observed to have an unusually long length of stay (259 days) and to have a positive test for colonization approximately mid-way through the hospital stay. To investigate the possible influence of a long-stay patient on other patients in the unit, we performed 16 sample simulations using actual entry/exit and observation times but simulation colonization and transmission events. The disproportionate effect of this long-stay patient is apparent in 16 sample simulations shown in S1E Appendix.

In 7 of 16 simulations, the long-stay patient becomes colonized with a mean time to colonization of 128 days, essentially the midpoint of the patient’s stay, and an average time spent uncolonized of 130 days. The mean total prevalence of simulations in which the long-stay patient became colonized was 526 colonized patient-days. In 3 of the 7 simulations in which the long-stay patient becomes colonized, colonization occurs before the mid-point of the patient’s stay after 38, 20, and 42 days, respectively.

After excluding the days colonized (if any) of the long-stay patient from each simulation’s total prevalence, there was a mean of 396 colonized patient-days over the 7 simulations with a colonized long-stay patient and a mean of 275 colonized patient days over the 9 simulations in which the long-stay patient remained uncolonized, resulting in a difference of 120 colonized patient days between simulations with colonized versus non-colonized long-stay patients. This resulted in an increase of 44%. The times and duration of the simulated long-stay patient’s colonization (if any) are shown in S1D Appendix.

### Total prevalence

Colonization pressure, or the fraction of colonized patients in a hospital unit, is one risk factor for a patient becoming colonized or infected [25, 74]. We can estimate the mean colonization pressure, or the fraction of colonized patient-days, with the theoretical total prevalence calculated from the reduced state model (Fig 5) and input parameter values. Thus, we can determine the expected fraction of colonized patient-days over the course of the study at particular parameter values. The theoretical total prevalence calculated from the best-fit parameters was 0.07 of the total colonized patient-days of the entire active surveillance study (the **bold star** in all panels). Thus, for the case where active surveillance was performed in a hospital unit of 13 beds and in which complete occupancy is assumed, the model predicts that 7% or about 380 of 5421 total patient-days would be colonized.

**Fig 7:**
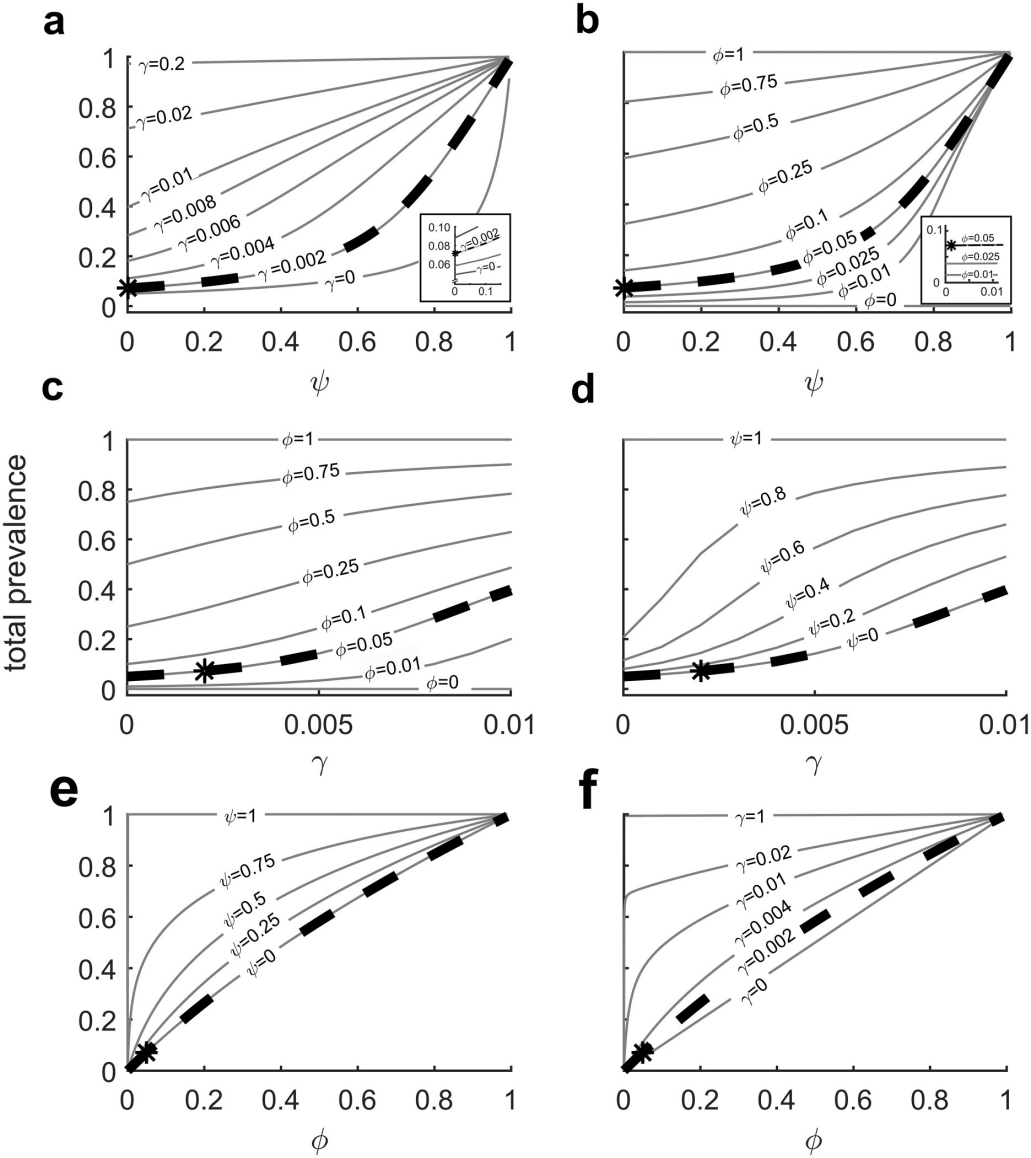
Predicted total prevalence of colonization in a 13-bed hospital unit as a function of the model parameters *γ*, *ϕ*, and *ψ*. Each panel shows the predicted total prevalence as a function of one model parameter for selected values of a second model parameter, with the third parameter held constant at the best-fit value for the rehabilitation data (inferred using the full detailed model inference method). The bold star (*) shows the point at which all parameters have the inferred best-fit values (*γ* = 0.002029/day, *ϕ* = 0.0497, and *ψ* = 0.000946). The bold dashed curve shows the case in which two of the three parameters have their best-fit inferred values while the third parameter is varied. The panels show total prevalence as a function of (a) *ψ* and *γ*, (b) *ψ* and *ϕ*, (c) *γ* and *ϕ*, (d) *γ* and *ψ*, (e) *ϕ* and *ψ*, and (f) *ϕ* and *γ*. The insets in panels (**a**) and (**b**) show enlargements of the area near the origin. In the inset of panel (**a**), the values of *γ* for the contours from bottom to top are *γ* = 0, 0.001, 0.002, 0.003. Enlarged versions of all panels are available in S1M Appendix.

Furthermore, we can estimate the fraction of total colonized patient-days over the course of the study that are attributable to various mechanisms of colonization. Fig 7 shows how the total prevalence would vary if one parameter was fixed at the best-fit parameter value and the other two parameters were varied. (Expanded versions of each plot are available in S1M Appendix). For example, in Fig 7f, *ψ* is fixed at *ψ* = 0.0009, and the plot shows how total prevalence varies with increasing *ϕ* and *γ*. The bold star shows the total prevalence (0.07) with all parameters at the best-fit values (*γ* = 0.002, *ϕ* = 0.05, and *ψ* = 0.0009). Because prior-to-new transmission was found to be effectively zero, in absence of patient–patient transmission, the total prevalence increases linearly with and is equal to the pre-existing prevalence, shown as the linear diagonal line for *γ* = 0 in (Fig 7f). Thus, given a theoretical total prevalence of 7%, with *ϕ* = 0.05, 5% of mean colonization-days can be attributed to pre-existing colonization; the remaining 2% can be attributed to patient–patient transmission, with prior-to-new colonization contributing a negligible amount. Note that while *ϕ* describes the fraction of entering patients that are colonized, those patients will remain colonized for the entirety of their stay, so the colonized patient days will compose 5% of total patient days.

Fig 7a shows the effect of prior-to-new patient colonization probability (*ψ*) on total prevalence at various levels of patient–patient colonization (*γ*) but fixed pre-existing colonization (*ϕ* = 0.05). These results suggests that the total prevalence is not very sensitive to the effect of prior-to-new patient colonization at the best-fit parameter values (bold star). However, as the patient–patient transmission rate increases, the effect of prior-to-new bed-patient transition becomes more pronounced, with the greatest slope (increase in total prevalence with increasing *ψ*) occurring with *γ* = 0.008 to 0.01.

Fig 7b shows the relationship between the prior-to-new patient colonization probability (*ψ*) and the total prevalence with varying pre-existing colonization probabilities (*ϕ*) but a patient–patient transmission rate fixed at the best-fit value(*γ* = 0.002/day per colonized/uncolonized patient pair). Like Fig 7a, the total prevalence is quite insensitive to changes in *ψ* at the best-fit values of *ϕ* = 0.05 and *γ* = 0.002/day per colonized/uncolonized patient pair, but it has the greatest increase with increases in *ψ* (has the greatest slope) at *ϕ* = 0.5. However, at *ϕ* = 0 or *ϕ* = 1, in which no patients or all patients are colonized, respectively, the total prevalence is constant at 0 or 1, regardless of the prior-to-new patient transmission probability.

Fig 7c shows the total prevalence at different values of the patient–patient transmission rate (*γ*) and pre-existing colonization (*ϕ*), with *ψ* fixed at the best-fit value of *ψ* = 0.0009. With zero (*ϕ* = 0) or all (*ϕ* = 1) patients entering colonized, the patient–patient transmission rate is irrelevant because the total prevalence is, respectively, 0 or 1. At the best-fit parameter values (bold star), the total prevalence is about 0.07, but doubling the patient–patient transmission rate to *γ* = 0.004/day (per colonized/uncolonized patient pair) increases the total prevalence to 0.11, an increase of 0.04. However, if the pre-existing colonization *ϕ* were to double from *ϕ* = 0.05 to *ϕ* = 0.10, the total prevalence would increase to 0.14. At *ϕ* = 0.10, doubling *γ* to *γ* = 0.004/day increases the total prevalence to 0.20. At *ϕ* = 0.25, doubling *γ* increases the total prevalence from 0.32 to 0.48, a much greater absolute increase of 0.16. Thus, modest increases in pre-existing colonization will magnify the effects of small changes in patient-patient transmission.

Fig 7d shows the effect of patient–patient transmission and prior-to-new colonization on total prevalence with pre-existing colonization fixed at the best-fit value of *ϕ* = 0.05. At the best-fit parameters (bold star), changing the patient–patient transmission does not greatly increase the total prevalence, but as the prior-to-new patient colonization probability increases, small increases in patient–patient transmission are magnified and subsequently have a greater effect on total prevalence.

Fig 7e shows the effect of prior-to-new patient colonization on total prevalence with fixed patient-patient transmission at the best fit value of *ϕ* = 0.05. The bold dotted line showing the total prevalence at the best-fit prior-to-new patient colonization (*ψ* = 0.0009) is essentially the diagonal line, suggesting that the amount of total prevalence attributable to prior-to-new bed-patient colonization is effectively zero.

Fig 7f, as discussed above, shows the relationship between pre-existing prevalence (*ϕ*) and total prevalence at different patient–patient transmission rates, with prior-to-new colonization fixed at the best fit value of essentially zero (*ψ* = 0.0009). The diagonal line at *γ* = 0/day shows that total prevalence equals pre-existing prevalence if there is no patient–patient transmission. The increasing values of *γ* above the diagonal show the effect of patient–patient transmission on total prevalence. At the best-fit parameter values (bold star), the increase in total prevalence contributed by patient–patient transmission is small. However, as the patient–patient transmission rate increases, the fraction of total prevalence contributed by patient–patient transmission becomes greater. Additionally, at very high values of *γ*, the initial slopes become nearly vertical, suggesting that colonization spreads rapidly (an outbreak caused by entry of an initially colonized patient) and the majority of total prevalence is attributable to patient–patient transmission.

## Discussion

In this paper, we developed a stochastic mathematical susceptible-infective (SI) model of bacterial transmission within hospital units that uses the full detailed state model (FDM). The FDM tracks individual patient colonization statuses, and it includes three mechanisms of colonization: patient–patient transmission, prior-to-new patient colonization, and pre-existing colonization. The FDM employs a stochastic hybrid continuous-time/discrete-time Markov model so that it can incorporate arbitrary times for patient exit/entry and testing. We used the FDM to develop an inference method that incorporates active surveillance data and hospital census data to distinguish parameters for three routes of transmission, including from prior to new patients. This inference method allows incorporation of incomplete test results and calculates the total probability of *all* possible sequences of states consistent with observation. We also compare the FDM with a reduced state model (RSM) similar to previously published models for in-hospital transmission [9, 32, 34, 37, 38, 41–43, 46, 47, 75] that track the *number* of colonized or infected patients rather than the status of each individual patient.

In the context of previous stochastic models [31] of patient colonization within hospital units (compared in S1 Table), this work incorporated five major modeling choices: 1) using a detailed rather than reduced state model (i.e., tracking individual patient statuses versus counting the number of colonized patients); 2) modeling evolution of states with a hybrid of discrete-time and continuous-time approaches; 3) incorporating three mechanisms of patient colonization, including prior-to-new (bed-patient) colonization; 4) allowing for incomplete observations and patient turnover at arbitrary times; and 5) calculating likelihood directly while incorporating partial observations.

The majority of models of in-hospital transmission use the reduced state, counting the number of individuals in each compartment [32, 34, 37, 38, 41–43, 46–48, 75]; the full detailed state is used far less frequently in forward model formulation [33] although individual patient statuses may be tracked during MCMC algorithms [34, 35, 39]. Of note, the general model of López-García & Kypraios [36], if formulated with each patient as a compartment, would be equivalent to using a detailed state model with individual transmission rates (which would not be able to be represented as a reduced model). Although the reduced state may be sufficient for inferring patient-patient transmission rates—and in fact, the “compression” method described in S1N Appendix effectively transforms the full detailed state to the equivalent reduced state *between* events—the full detailed model is required at exit/entry and test events to track individual patient colonization statuses, which improves the model’s ability to uniquely identify parameters for bed-patient and pre-existing colonization. The reduction of the full detailed model is equivalent to the approach of graph-automorphism driven “lumping” by Simon and Kiss, who show that the exact solution of a continuous-time Markov Chain individual-based epidemic model converges to that of an ordinary differential equation-based mean field (e.g. reduced) model [76]. However, although the full detailed model and reduced model can be shown to be equivalent in formulation of the *forward* model (both statistically and in simulation), for purposes of *inference* they are not equivalent. The full detailed model performs better because it retains information about individual patients that can distinguish pre-existing or prior-to-new patient colonization from patient-patient colonization.

Most stochastic hospital transmission models use either continuous time [37, 38, 41–43, 48] or discrete time [32, 33, 40, 47] Markov models. To our knowledge, this model is the first in-hospital transmission model to incorporate a hybrid continuous-time/discrete-time approach, and also the only model that explicitly incorporates bed-patient colonization, although previous models included an environmental transmission component [36]. However, like many other groups [77, 78], we incorporated transmission from infected or colonized patients to susceptible patients [29] and included pre-existing colonization [32, 38, 42, 46–48].

Although multiple models incorporate incomplete observations [32, 33, 34, 42, 75], our approach is most similar to previous efforts that incorporated arbitrarily timed tests [34]. Similar to previous methods, we assume perfect specificity [35, 39], but unlike other groups, we do not directly estimate parameter sensitivity [34, 35, 39, 40, 47]. We instead use a “once colonized, always colonized” approach to testing, assuming that even if colonization-positive patients test negative, they nevertheless remain colonized because decolonization occurs on a timescale of months to years [67] and patients can retest positive after one [19] or even multiple [67] negative swabs.

We build on previous work directly calculating the likelihood of continuous-time Markov models [38, 41] and combine it with other approaches such as a discrete-time method that uses observations to reduce the number of possible states [32] and incorporates exact times of testing and turnover [36, 35, 39, 46–48]. A hybrid continuous-time/discrete-time formulation allows the flexibility to incorporate events that occur at varying time intervals while also accounting for events that are modeled as occurring instantaneously, such as patient exit/entry and exposure to a potentially contaminated bed. This eliminates a parameter and avoids assumptions that turnover times have a constant rate [30, 38] or exponential distribution [36]. The technique of compressing the continuous-time portions of the model incorporates techniques similar to previous work reducing the dimensionality of an exact stochastic SIS epidemic network with 2^n^ possible states [71] and “lumping” over a complete graph using automorphism [76]. The overall approach to calculating likelihood is generalizable to any Markov model that can ultimately be represented as a series of transition probability matrices. Although direct calculation of the likelihood of all possible permutations of events consistent with observed data is difficult or even “intractable” [39, 47], the problem is simplified when represented in matrix form (similar to previous work [32]): test results reduce the number of possible states and thus the dimensions of the sub-matrices representing the probability of transitions from event to event. Unlike approaches incorporating MCMC augmentation of data with “guesses” about the times of colonization events [34, 35, 39], the likelihood of all observed test results and unobserved colonizations is already incorporated into the likelihood and does not require additional computation during parameter estimation. However, use of MCMC methods could allow incorporation of prior information about parameters.

### Mechanisms of colonization

To our knowledge, this model is the first in-hospital transmission model to explicitly include prior-to-new transmission [79], although many previous stochastic models of in-hospital transmission have included pre-existing colonization and patient–patient transmission [32, 34, 38, 41, 48]. Although antibiotic exposure was found to be a risk factor for CRE colonization and infection in long-term acute care facilities [2, 80] and hospitals [18, 81–83], patients were generally not admitted into the rehabilitation unit while on intravenous or parenteral antibiotics, so we choose to assume that all patients have equal susceptibility to bacterial transmission. We also assumed that patient-patient transmission rates are linearly proportional to the number of colonized-uncolonized patient pairs, consistent with findings that the odds ratio of transmission in long-term acute care hospitals increased approximately linearly with increased colonization pressure [74] and that a linear patient-patient transmission model fit better than a non-linear model for medical hospital units [35]. Our model did not include background colonization, as two previous modeling efforts found no significant difference in model output or goodness-of-fit between mathematical models that did or did not include background colonization [35, 39]; one even found a slight preference for a no-background model in hospital medical units [39]. The true background transmission rate is unclear as most datasets included only 1-2 colonization mechanisms without prior-to-new patient colonization, but the fraction of acquired colonization attributable to mechanisms other than patient-patient transmission may range from none [39] to approximately half [84] or three-quarters [39, 68]. In our model, inclusion of a background colonization rate would be equivalent to adding a random noise term for sources including spontaneous emergence of resistance [85], unmasking of existing colonization by antibiotics [74], and transmission from outside sources such as visitors or equipment [47]. De novo creation of resistant strains seemed unlikely as the dataset used showed primarily clonal transmission of the ST258 strain of *K. pneumoniae* [25]. Unmasking of colonization was also less likely in a rehabilitation unit than in an intensive care unit, as the patients were required as a condition of admission to be able to participate in their care, and thus were rarely on parenteral or intravenous antibiotics. Plasmid movement was also not included as it occurs infrequently [86]) and therefore likely does not significantly affect results given the short average patient length of stay. However, if the model was applied to a dataset tracking mobile genetic elements for resistance [1] rather than a specific strain of CRE, transmission could be interpreted as the spread of those genetic elements [87, 88] from patient to patient.

### Non-equivalence of the full and detailed states for inference

The classic SIR compartment model represents the state of a population as the numbers of susceptible, infective, and resistant patients. Most models of bacterial spread within hospital units [32, 38, 41] count only susceptible and infective patients and use longitudinal prevalence as the state of the unit, with the exception of one model of *S. pneumoniae* carriage within families, which tracked the infection statuses of individual members within households [33]. As shown in Fig 5, we compared the FDM against a simpler continuous-time, reduced-state model (RSM) in which only the prevalence, i.e., the number of colonized or uncolonized patients, was considered (similar to a susceptible-infected-susceptible model). In the RSM, the same three routes of transmission were included, but turnover was assumed to occur at a constant rate *β* which was estimated from patient census data. Surprisingly, although the two models are equivalent for the forward problem of creating a simulation using given input parameters, the FDM and RSM are not equivalent for the inverse problem of parameter inference. Using the same parameters and initial state of the hospital unit, both the FDM and RSM give equivalent statistics and predictions about future states, such as the distribution of colonized patients at steady state. In the context of the inverse problem, however, the FDM has a greater ability to distinguish and uniquely identify parameters for patient-patient transmission versus pre-existing colonization from data.

For example, consider a scenario in which hospital unit testing occurs after each of three patient turnover events. At each test time, there is a single colonized patient within the unit (the reduced state), but inspection of the full detailed states could show different scenarios, such as a series of three patients in the *same* bed being colonized (001, 001, 001) or three patients in *different* beds being colonized (001, 100, 010). The former scenario suggests prior-to-new colonization, but the latter suggests pre-existing colonization. However, with the reduced model, both scenarios yield an identical series of states (1, 1, 1), discarding location information that could help distinguish the colonization mechanisms. This may account for some of the difficulties of past modeling efforts to uniquely identify parameters for patient–patient transmission versus spontaneous colonization [38] or pre-existing colonization, as aggregating data into counts diminishes the available information [41].

Cooper et al. used a hidden Markov model that required at least 40 months of contiguous data with at least 1 case per month. For a series with fewer than 20-30 observations, maximum likelihood estimates may fail to converge, and collinearity between parameter estimates can lead to identifiability problems. This may result from a loss of information that occurs with data aggregation [41]. We observed a similar effect in comparing the ability of FDM and RSM models to recover input parameters from “observations” sampled from simulated data created with those parameters and the identical test scheme used in hospital surveillance. The difference between input and recovered parameters for each inference method reflects the effects of incomplete information—including encoding state as the point prevalence (the number of colonized patients at a given test time)—and randomness.

Usage of the full detailed state reduces the problem of parameter collinearity or non-identifiability. The FDM model could easily distinguish parameters for patient–patient transmission and pre-existing colonization in simulated data, and its estimate of the prior-to-new-patient colonization probability was improved compared to that of the RSM, which consistently overestimated *ψ*, as shown in Table 1. The FDM model had a smaller estimated error range than the RSM model, although the estimate of *ψ* still had large uncertainty, likely because of the small or near-zero parameter value (see additional comments in “**Epidemiological Relevance**” below). In general, the overall parameter estimation error of the FDM (0.0005) was much smaller than that of the RSM (0.12). These results show that the inference method can in fact recover estimated parameter values reasonably close to the parameter values used to create the simulated data. The sum of squares error (SSE) for the FDM is smaller than that of the RSM, suggesting that parameter estimation for the FDM is better than for the RSM. Thus, tracking individual patient information of the detailed state can improve parameter identification for different mechanisms of transmission.

### Computational challenges of partial observation

The approach of looking at all particular states consistent with observations has been applied to reduced state models [32, 37]. Although conceptually similar, calculation of the likelihood function is far more difficult in the FDM case because the number of states consistent with an observation increases *exponentially* with the number of unobserved patients. If all beds are tested at a given observation time, there is only one possible state, but in reality, most surveillance studies do not have 100% compliance and test only *some* beds in a unit at a given observation time. Testing reduces the number of possible hospital unit states conditioned on the test results: if *m* of *n* patients in the unit are tested, then there are 2^*n*−*m*^ (FDM) or 2(*n* − *m*) + 1 (RSM) possible states consistent with the test results at that observation time. The number of possible states was reduced even further by using the assumption of “once colonized, always colonized” (similar to previous approaches [32, 37]). A number of groups have used MCMC methods to sample over the space of states with times of colonization consistent with test results [34, 35, 39, 47].

Despite the exponentially larger number of possible states in the detailed model, the inference method presented in this paper still permits exact likelihood calculation that takes into account all possible sequences of hospital unit states consistent with partial observations, although this may be computationally expensive for larger units. The likelihood calculation is generalizable to any Markov model that can be reduced to a series of probability transition matrices. (In this paper, we transform the rate matrices from the continuous-time portions of the hybrid model into probability matrices for state changes over unequal time intervals). Likelihood calculations were made computationally tractable through the use of matrix “compression” (S1N Appendix) and a method for exact matrix exponentiation based on the Jordan form (S1O Appendix). These techniques drastically reduce the time and computer memory needed for calculating the multiple large matrix exponentials required for the continuous-time portions of the model between exit/entry events. After implementing these changes, calculation of the likelihood of a single set of parameters over the study period data (weekly sampling of 13 beds over 1 year) decreased from hours to approximately 7 minutes using lightly optimized MATLAB code on the Center for Health Informatics and Bioinformatics cluster. Use of a compiled rather than interpreted language or a system with additional random access memory (RAM) would likely decrease computation time significantly.

### Epidemiological relevance

Colonization pressure is the proportion of patients already colonized or infected with pathogen [16]. In this study, we define it as the fraction of colonized patient-days of total patient-days in a hospital unit for a given period of time such as a month or year (as opposed to point prevalence, estimated as the fraction of positive tests at a given testing time [37]). This is equivalent to the theoretical total prevalence from Eq 5, the probability that at least one patient is colonized. (Note that the total prevalence is not equivalent to the probability of pre-existing colonization, which is the fraction of *incoming* patients that are colonized). Estimating the colonization pressure can be problematic if not all patients were tested and/or if colonized patients are tested at a different frequency than untested patients; in this work, testing is assumed to be arbitrary rather than random, so using clinical test results for patients suspected of having colonization or infection will not bias the results. Using best-fit parameters from the FDM, we were able to estimate the total prevalence at different parameter values and also estimate the relative contributions of the different mechanisms of colonization to the total prevalence. The theoretical total prevalence was estimated to be approximately 7%, similar to an estimate of 5.4% for New York City hospitals [25]. We were able to estimate the total prevalence attributable to patient–patient transmission (2%) over the baseline pre-existing colonization (5%). The best-fit patient-patient transmission rate of 0.002 per colonized-uncolonized patient pair per day, or 2 per 1000 colonized-uncolonized patient pairs per day, is consistent with the work of Hilty et al., which found a rate of between 0.0006 and 0.002 per colonized uncolonized patient-pair per day for extended-spectrum beta-lactamase *K. pneumoniae* [68], assuming that the colonized study patients had been exposed to only one index case at a time. The estimate of pre-existing colonization (e.g., colonization of patients prior to entry into the hospital unit) is consistent with an estimate of community CRE prevalence of 5.6-10.8% in the United States [10] and observations that many patients were already colonized upon entry [1, 89]. Patients with pre-existing colonization may have become colonized during previous hospitalizations [67] (as was also previously found for VRE [90]), or within the community [68]. This suggests that infection control procedures within the rehabilitation unit should focus on identifying and isolating colonized patients on admission, and that improvements in handwashing [91] and other measures targeted at decreasing patient–patient transmission can only lower total prevalence to the baseline of pre-existing colonization. However, if pre-existing colonization prevalence increases, measures to reduce patient–patient transmission become increasingly important.

Our model found that the contribution of prior-to-new-patient colonization to total prevalence was effectively zero, suggesting that environmental contamination of CRE in the rehabilitation unit may be a lower-yield target for intervention. This is consistent with results from a study which showed that environmental contamination was a minor contributor to overall transmission, despite an increased risk of acquisition for patients admitted to rooms previously occupied by patients colonized by MRSA or VRE [79]. Our result differs from a of ICU colonization by *K. pneumoniae*, which showed that occupying a bed previously occupied by a colonized patient was a major risk factor for colonization (odds ratio 4.8) [26]. However, this may reflect a difference in illness severity of ICU patients compared to rehabilitation patients or local factors such as cleaning methods [92] and room layout. In the rehabilitation unit of this study, patients were frequently transported in their beds from other locations rather than being placed into the same bed as the previous patient. In this case, the prior-to-new-patient colonization parameter would only describe colonization from surfaces surrounding the bed (such as sink drains [93], ventilators [94], tables or curtains) that remained in the same location and may have had fewer opportunities for patient contact. This is consistent with a study of CRE environmental contamination that found that 75% of the contamination occurred in beds and 25% in the surrounding environment, although there was a large variation in the degree of environmental contamination between different patients and even the same patient at different times [95]. Thus, the poor estimation of *ψ* may have been a result of the rarity of event occurrence or a mismatch between model assumptions and hospital conditions; likely this estimate would improve in situations with fewer confounding factors or in situations in which prior-to-new-patient colonization occurred more frequently. The model does not account for prior-to-new patient transmission occurring at distant time intervals or via methods other than patient-bed-patient transmission, as one institution found delayed transmission between patients inhabiting the same room even months apart [96].

The simulation results suggest that patients with longer-than-average hospital stays may become reservoirs of transmission. These long-stay patients may contribute disproportionately to outbreaks by acting as a source for multiple patient–patient transmission events. Thus, targeting long-stay patients for additional screening and possible isolation or decolonization (if possible) may be a high-yield infection-control measure, as colonized patients with shorter stays have fewer opportunities for transmission. Furthermore, additional infection-control measures may be considered to prevent colonization of long-stay patients to protect *other* hospital patients.

### Other limitations

Our model identifies as “transmission” only cases in which patients are simultaneously present in the hospital unit for some length of time. For pathogens in which colonization or its detection might be delayed significantly, the model may not be appropriate.

Although use of the hybrid continuous-time/discrete-time model allows use of arbitrary exit/entry times and testing times, it is computationally expensive to calculate the likelihood function. The real “trade-off” in the choice to represent state as a scalar (the number of colonized/infected patients) versus vector (a list of the statuses of the patients in a unit) is that the size of the matrix increases linearly versus exponentially, respectively. However, the assumption that patients are equally likely to colonize or be colonized by any other patients within the unit may be unwarranted in hospital units with large numbers of patients. Thus, there will be an upper hospital unit size limit in applicability of this method because of the assumptions, regardless of the computational cost.

Additionally, some of the mathematical and computational techniques presented in this paper and its appendices exploit the specific structure and assumptions of our colonization and transmission model—in particular, the methods of matrix “compression”, factorization of *γ*, and use of Jordan form. Future modifications to the mathematical model that would eliminate of some of the symmetries in patient–patient transmission (such as incorporation of additional parameters for spatial transmission or random colonization) would prevent use of these matrix compression methods on the continuous-time portions of the model. However, removal of these symmetries, which make the matrix defective, will allow use of standard techniques for matrix exponentiation such as diagonalization.

We did not calculate test sensitivity for reasons described previously, but incorporation of sensitivity into the model either as part of the likelihood framework or via a Bayesian method would be a valuable future extension of the model.

### Conclusion

Although new tools such as KlebSeq [97] promise to make sequencing more widely available, hospitals likely will continue to have older methods of CRE detection in place. Our mathematical model and inference techniques provide a method for hospitals to estimate the fraction of colonization attributable to nosocomial transmission without need for sequencing, even with incomplete testing of incoming patients. (Should sequencing become available, data from individual strains or for particular resistance plasmids can be used as input, although there is some minimum amount of data required for parameter estimation). Because hospitals in the United States are penalized by the government for patient infections [98, 99], this method to distinguish pre-existing from hospital-acquired colonization may prove valuable, especially as it does not require additional resources and personnel for genetic sequencing [88] but can use existing testing methods. It is applicable to any nosocomial pathogen for which appropriate surveillance data exists and for which the mechanisms and assumptions of the models described in this paper are applicable. In particular, the model may be well-suited for describing MRSA, which colonizes the epithelium [100] and can be transmitted by direct skin and fomite contact [101, 102]. Other potential pathogens for which the model may be well-suited include *C. difficile*, *Pseudomonas* [102], and *E. coli*, which persist on contaminated surfaces in concentrations high enough for transmission [102, 103]. It may also be applicable to SARS-CoV-2. The model assumptions are less well-suited for pathogens that persist for shorter times on surfaces, for which person-person transmission or long-term carriage does not occur, and/or in which an extended non-infectious incubation time occurs. The inference method will work better for pathogens whose colonization prevalence falls in the intermediate range between non-existent and complete colonization.

In summary, the full detailed mathematical model and inference method enables estimation of parameters for three possible routes of colonization from active surveillance data. Using these parameters, we can estimate the importance of patient–patient transmission and effectiveness of interventions on colonization pressure, and we can also simulate realistic scenarios with actual patient exit/entry times. Crucially, the inference method allows direct maximum likelihood estimation of parameters from *all* possible states consistent with incomplete and nonrandom observations without need for MCMC techniques. We also demonstrate the utility of tracking the colonization statuses of individual patients for unique parameter identification. Incorporation of additional routes of colonization and transmission, including exogenous colonization from sources other than the patients (such as visitors), is an area for future research.

## Supporting information

S1 Appendix Mathematical methods. (ZIP)

S2 Appendix Data description. (ZIP)

S1 Table Comparison of mathematical models. (ZIP)

S1 Data Data used for analysis. (XLS)
