# Supplementary material for: A mathematical model and inference method for bacterial colonization in hospital units applied to active surveillance data for carbapenem-resistant enterobacteriaceae
Source: PLoS One. 2020 Nov 12;15(11):e0231754. doi: 10.1371/journal.pone.0231754 (PMC7660488; doi:10.1371/journal.pone.0231754)
Supplement: S1 Appendix — (ZIP) [file pone.0231754.s001.zip › S1_Appendix.pdf]

S1 Supplemental Materials: Mathematical Theory

Mathematical Theory: A Mathematical Model and Inference Method for Bacterial Colonization in Hospital Units Applied to Active Surveillance Data for Carbapenem-Resistant Enterobacteriaceae

Karen M. Ong, Michael S. Phillips, Charles S. Peskin

Appendix S1-A

A Method for Conversion of Binary Vectors to Integer Indices

For the purpose of creating transition matrices, it is convenient to convert binary state vectors (or equivalent bit strings) into integers representing the state of the unit. Each digit in the big string corresponds to a particular bed in the unit. However, because this particular model does not take into account the spatial relationships amongst beds or patients, it does not matter which bed a particular digit refers to as long as it is the same bed throughout time. Any method that assigns binary vectors to equivalent unique integers is appropriate. Here, we present one method of converting a binary vector b, which represents the state of an n-bed unit, to an integer j, where vector components b_k represent the colonization state of bed k, for $k = 1, 2, \ldots, n$:

$$j = \sum_{k=1}^{n} 2^{k-1} b_k \tag{1}$$

In order to invert the above relationship and convert the integer j back into the binary digits $b_1 \ldots b_n$, proceed as follows. First, set

$$b_1 = \mod (j, 2) \tag{2}$$

Then, for $k = 2, 3, \ldots n$, set

$$b_k = \mod \left(j - \sum_{\ell=1}^{k-1} 2^{\ell-1} b_\ell, 2 \right) \tag{3}$$

This is essentially a binary representation of integers, but we reverse the order of the bits so that the least significant bit comes first. In implementing this schema, we arbitrarily chose to have the digits in the binary vector correspond lexicographically with the bed location labels, which roughly correlate with geographic location. Using **Equation 1**, we represent the state of the full detailed unit as an integer that corresponds with a particular configuration of uncolonized and colonized patients within the beds of a hospital unit. (Note that for the reduced model, the integer representing a state of the unit corresponds with the *number* of colonized patients, resulting in a patient-patient transmission rate matrix with a single superdiagonal.) Given that integers can be arbitrarily

chosen to correspond with states of the hospital unit, it may be possible to assign states such that the patient-patient transmission matrix R is tridiagonal-by-blocks, allowing for additional methods of matrix exponentiation such as those described by Economou et al.[6]. Note that the integer index j has the range $0 \ldots 2^n - 1$ rather than $1 \ldots 2^n$, a convention that is non-standard for a matrix index. Because MATLAB does not allow 0 as an index, the indices were converted to $j' = j + 1$ for use in MATLAB.

Appendix S1-B

Stochastic Simulation of Patient Colonization at an Entry/Exit Event

Consider the z-th exit/entry event occurring at time t_z in bed k_z. Assume that we know the previous state vector $\mathbf{b}(t_z^-)$, the state of the hospital unit at the time the patient in bed k exits. Using simulation, we wish to determine the final state of the hospital unit after entry of a new patient into bed k and possible prior-to-new colonization.

In this section, for convenience, we will drop all z subscripts and write $\mathbf{b}^-$ for the initial state vector and $\mathbf{b}^+$ for the final state vector. Each component b_i describes the status the individual patient in bed $i \in \{1, ...n\}$. Here, k is the integer index of the bed in which turnover occurs. Assume that entering patients have a pre-existing colonization probability ϕ. Uncolonized patients entering a bed previously occupied by a colonized patient can become colonized with probability ψ (prior-to-new colonization). **Table A** summarizes the probabilities of transition between initial state b_k^- to b_k^+ for the bed in which turnover occurs.

	$b_k^+ = 0$	$b_k^+ = 1$
$b_k^- = 0$	$1 - \phi$	ϕ
$b_k^- = 1$	$(1 - \phi)(1 - \psi)$	$(1 - \phi)\psi + \phi$

Table A. Transition probabilities for the bed (k) in which turnover occurs during an exit/entry event. Here, b_k^- is the initial state of the bed (the state of the exiting patient) and b_k^+ is the final state of the bed (the state of the new patient after entry and possible prior-to-new colonization).

Notice that the final state of the k-th *bed* determines the final state of the entire hospital *unit* because all other patients remain in the same state. Thus, the final state of the hospital unit is a vector $\mathbf{b}^+$ in which the final state of the bed in which turnover takes place (b_k^+) is determined by the probabilities in **Table A**, but all other vector components remain in the same status as they were initially ($b_\ell^- = b_\ell^+$ for $\ell \neq k$). The final state of bed k after an exit/entry event depends on the state of the exiting patient (b_k^-) and the colonization status of the entering patient (S) before possible prior-to-new patient colonization. If the entering state of the new patient in the k-th bed is unknown, we choose S randomly with probability ϕ. Otherwise, we use the given value of S to determine the final status of the bed, as follows.

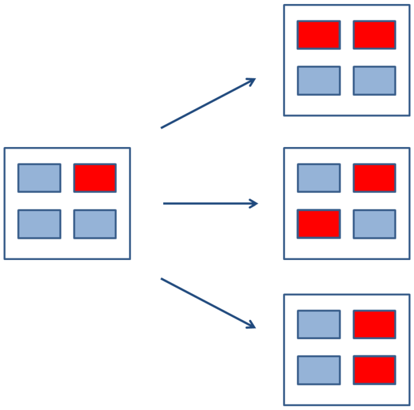

Figure A. Event-driven simulation method for a continuous-time Markov chain. In this model, it is used to simulate instances of patient–patient transmission occurring between exit/entry events.

If the exiting patient is uncolonized ($b_k^- = 0$), no prior-to-new patient colonization can occur. Thus, the final status of bed k is completely determined by the status of the incoming patient, so $b_k^+ = S$. If the exiting patient was colonized ($b_k^- = 1$) and incoming patient is colonized ($S = 1$), the final status of bed k is simply $b_k^+ = S$. If the exiting patient was colonized ($b_k^- = 1$) and the new patient is uncolonized upon entry ($S = 0$), then there is some probability ψ of prior-to-new patient colonization. To simulate this, we set $b_k^+ = 1$ with probability ψ, and $b_k^+ = 0$ with probability $1 - \psi$.

Appendix S1-C

Event-Driven Simulation of Patient–Patient Transmission Between Exit/Entry Events

We use event-driven simulation[7, 8, 9] to simulate the occurrence of patient–patient transmission events, as shown in (**Fig A**). Consider a system in state i that can transition to one of N possible future states j after a random time interval T. We wish to simulate events on the time interval (t_{z-1}, t_z). Given that the system is in state i (an integer corresponding to the binary state vector), we wish to determine the next state $j \in \{1, 2, \ldots, N\}$ given a sequence of transition rates r_{ij}. (An example of possible transitions is shown in **Fig B**.)

We use event-driven simulation to determine when and where the next patient–patient transmission event will occur during a non-turnover interval. Consider the time interval between exit/entry events at time t_{z-1} and time t_z (for $z \in \{1, 2, \ldots, Z\}$). We simulate instances of patient–patient transmission events using event-driven simulation on the time interval (t_{z-1}, t_z) with rates drawn from the rate matrix R (from **Equation 7**). Assuming an initial state i at the start time, we find the time and state of the next event using the procedure

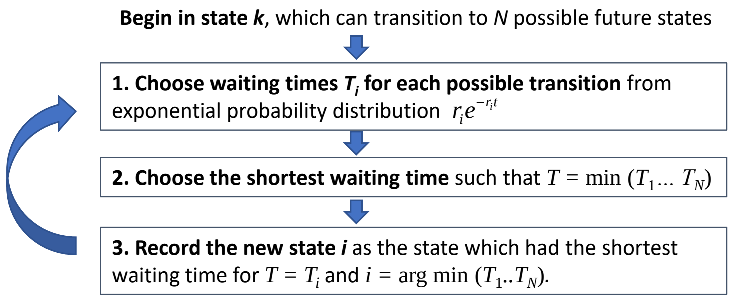

Figure B. An example of possible transitions to future states based on the previous state.

in **Fig A** with transition rates r_{ij} for all future states j. (This is equivalent to using a single row of rates from R for initial state i; any impossible transitions will have rate $r_{ij} = 0$). The procedure stops when it generates an event time $T > t_z$. We then ignore the transition that would have occurred at time t, and we use the state before that transition as the state of the system at time t_z^-. An example of choosing the next state based on the shortest waiting time is shown in **Fig C**.

Creating a simulation for a sequence of events Given input parameters γ, ϕ, and ψ; a list of $Z-1$ exit/entry events at arbitrary times $T = \{t_1, \ldots, t_{Z-1}$ that occur in beds $K = \{k_1, k_2, \ldots, k_Z\}$; and, optionally, a list of arbitrary states $S(z)$ of the entering patients at time t_z, we can create a simulation of the hospital unit for the period t_0 to t_Z by using the discrete-time matrix to simulate exit/entry events and the continuous-time patient-patient transmission rate matrix to simulate bacterial transmission between exit/entry events.

First, we find patient–patient transmission events for the time interval $t = t_1 - t_0$ for the hospital unit, which has an initial state $\mathbf{b}(0)$ that may include both colonized and uncolonized patients. After the last patient–patient transmission event in that interval (if any), the hospital unit retains its status until the first patient turnover event at time time t_1^-, at which time the k_1-th patient exits. We then simulate an exit/entry event on the interval t_1^- to t_1^+ (that is, at the time t_1) to find the status of bed k_1 after entry of a patient with colonization status $S(1)$ and possible prior-to-new patient transmission. For the non-turnover interval t_1^+ to t_2^-, we simulate patient–patient transmission events until we reach the next entry/exit event. We then repeat this procedure, alternating methods for determining patient–patient transmission between intervals and for determining the final state after an exit/entry event. Finally, after determining the state b_{Z-1}^+

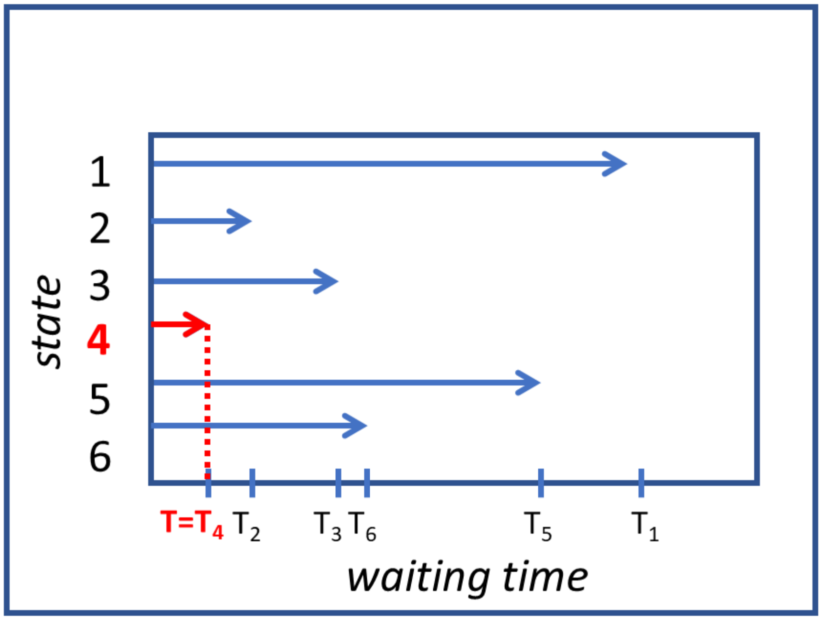

Figure C. An example of choosing the next state j based on the shortest waiting times. Here, waiting times for transitions to six possible future states are chosen from an exponential probability density $r_{ij}e^{-r_{ij}t}$, given the transition rates r_{ij}. The larger the rate r_{ij}, the shorter the expected waiting time, although for any given instance the actual waiting time may vary because of randomness.

for the last exit/entry event at time t_{Z-1}, we finish with one last patient–patient transmission simulation for the interval t_{Z-1} to t_Z.

Appendix S1-D

Results from Simulation of Hospital Unit Colonization and Transmission

Table B shows results from 16 sample simulations performed using parameters estimated from the active surveillance dataset (**Appendix S1-E**). The table highlights the effects of a long-stay patient on total colonized patient-days. All simulations were performed with the exit/entry times and locations from actual census data from the rehabilitation unit. Note that the sum of the time to colonization and time colonized is equal to the length of stay, so the sums of the numbers in the last two rows are constant (as all simulations used identical exit/entry schemes, so the length of stay of the long-stay patient did not vary).

Appendix S1-E

Sample Full Detailed Model Simulations

Fig D shows a sample of 16 full detailed model simulations that were created using the input parameters $\gamma = 0.00203$/day per colonized/uncolonized patient pair, $\phi = 0.049700$, and $\psi = 0.000946$. The vertical hashes show the times of exit/entry, the thin horizontal lines show that patients are uncolonized, and the thick red bars show when patients become colonized. Colonization caused by transmission is marked with a small cross above the red bar.

Appendix S1-F

Construction of the Entry/Exit Transition Matrix

In this appendix, we describe the construction of the exit/entry transition probability matrix. Each entry within the matrix describes the probability of transition from the initial state of the hospital unit at the time of a patient's exit to the final state of the hospital unit after entry of a new patient, contact with the bed, and possible prior-to-new patient colonization. Because we assumed that turnover occurs instantaneously, no time elapses between the initial and final state of this matrix.

Consider a hospital unit in which exit/entry events occur at times t_z for $z \in \{1, 2, \ldots, Z-1\}$ and the final observation occurs at time t_Z. At time t_0, the initial state of the hospital unit is the binary vector $\mathbf{b}(t_0)$. During an exit/entry event, a patient in bed k leaves at time t_z^- and is replaced by a new patient at time t_z^+. The overall state of the hospital unit changes from $\mathbf{b}(t_z^-)$ to $\mathbf{b}(t_z^+)$. Notice that only the k-th element of $\mathbf{b}$ changes because of the replacement of patients in bed k; the other elements b_ℓ for $\ell \neq k$ remain the same. To simplify notation when considering a single exit/entry event, we will drop the subscript z's and write the initial state vector as $\mathbf{b}^-$ and final state vector as $\mathbf{b}^+$.

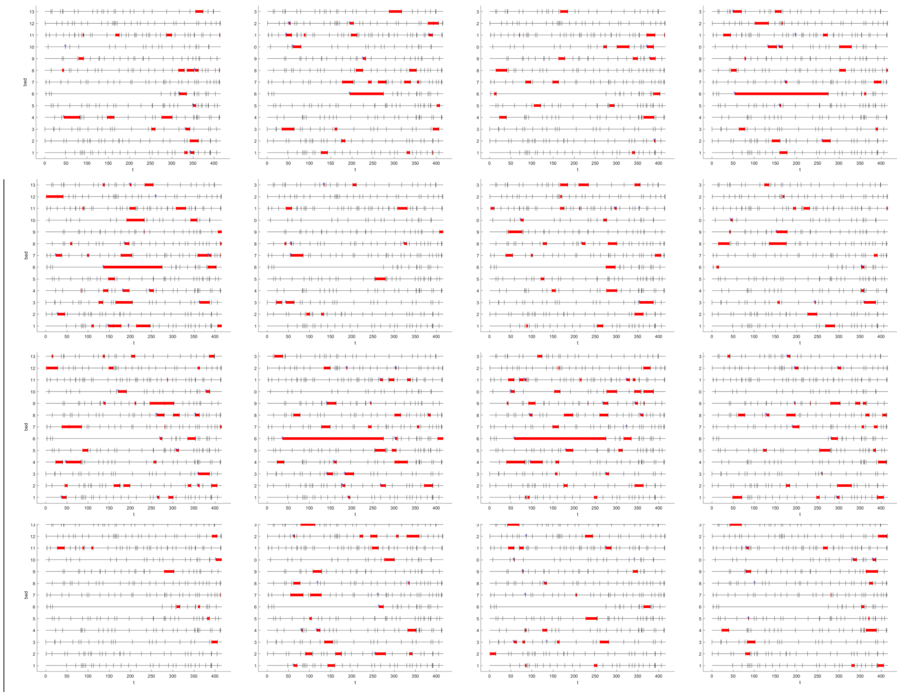

Figure D. Sample full detailed model simulations created using the input parameters $\gamma = 0.00203$/day per colonized/uncolonized patient pair, $\phi = 0.049700$, and $\psi = 0.000946$. The vertical hashes show the times of exit/entry, the thin horizontal lines show that patients are uncolonized, and the thick red bars show when patients become colonized. Colonization caused by transmission is marked with a small cross above the red bar.

**Trial**	**Colonized Patient-Days**	**Days to Colonization (Long-Stay Patient)**	**Days Colonized (Long-Stay Patient)**
1	257.502		
2*	407.923	178.525	80.376
3	285.527		
4**	516.404	37.704	221.197
5**	653.717	119.752	139.149
6	170.209		
7	336.714		
8	241.730		
9*	474.943	253.349	5.553
10**	583.141	19.858	239.0437
11**	662.541	42.0714	216.83
12	329.893		
13	402.953		
14*	385.148	247.6268	11.275
15	223.964		
16	232.882		

Table B. Results for total colonized patient-days overall and for the long-stay patient from 16 full detailed model simulations created using the input parameters $\gamma = 0.00203$/day per colonized/uncolonized patient pair, $\phi = 0.049700$, and $\psi = 0.000946$. In the table, * indicates the long-stay patient became colonized, ** indicates that the long-stay patient became colonized before mid-point of stay.

Let $M(S; k, \phi, \psi)$ be the general transition probability matrix for an exit/entry event in the k-th bed and in which the entering patient has a colonization status S. Here, S is a random variable that takes on the value 1 with probability ϕ (the pre-existing prevalence) and 0 with probability $1 - \phi$. The probability of a prior-to-new patient colonization event is ψ, and the probability that prior-to-new colonization does not occur is is $1 - \psi$. In some cases, the entering patient's status is known to be uncolonized ($s = 0$) or colonized ($s = 1$). The elements of M (probabilities) are $M_{\mathbf{b}^-\mathbf{b}^+}$, where $\mathbf{b}^-$ and $\mathbf{b}^+$ are the binary vectors (which correspond to appropriate integer indicies by a method such as that described in **Appendix S1-A**) for the initial and final states, respectively.

The matrix elements $M_{\mathbf{b}^-,\mathbf{b}^+}$ describe the probability of transition between state $\mathbf{b}^-$ and state $\mathbf{b}^+$ during a replacement event, given that the k-th patient exits with state b_k^-, the incoming patient either has known status S or has a pre-existing colonization probability ϕ, and the final state of the k-th bed is b_k^+ after possible prior-to-new patient transmission. The transition probabilities are nonzero only for state transitions in which the other, non-exiting patients in the hospital unit keep their colonization status through the transition. Any transitions in which more than one bed changes state have zero probability. For convenience in future sections, we will drop the arguments and designate the specific matrix $M(S; k, \phi, \psi)$ that corresponds with a replacement event at time t_z as $M(z)$. If the colonization status of the incoming patient is known, the entries of matrix M are as follows:

$$M_{\mathbf{b}^-,\mathbf{b}^+}(s; k, \phi, \psi) = \begin{cases} 1-s & b_k^- = 0,\ b_k^+ = 0 \text{ and } b_\ell^- = b_\ell^+ \text{ for } \ell \neq k \\ s & b_k^- = 0,\ b_k^+ = 1 \text{ and } b_\ell^- = b_\ell^+ \text{ for } \ell \neq k \\ (1-s)(1-\psi) & b_k^- = 1,\ b_k^+ = 0 \text{ and } b_\ell^- = b_\ell^+ \text{ for } \ell \neq k \\ (1-s)\psi + s & b_k^- = 1,\ b_k^+ = 1 \text{ and } b_\ell^- = b_\ell^+ \text{ for } \ell \neq k \\ 0 & \text{otherwise} \end{cases} \tag{4}$$

Here, $b_k^- \in \{0, 1\}$ is the k-th element of the state vector $\mathbf{b}^-$ and corresponds to the colonization status of the departing patient in the k-th bed upon exit; similarly, b_k^+ corresponds to the status of the entering patient in the k-th bed after entry and possible prior-to-new patient colonization.

If colonization status of the entering patient is unknown, the probability that the entering patient is colonized is ϕ, and the probability that the entering patient is not colonized is $1 - \phi$:

$$M_{\mathbf{b}^-,\mathbf{b}^+}(S; k, \phi, \psi) = \begin{cases} 1-\phi & b_k^- = 0,\ b_k^+ = 0 \text{ and } b_\ell^- = b_\ell^+ \text{ for } \ell \neq k \\ \phi & b_k^- = 0,\ b_k^+ = 1 \text{ and } b_\ell^- = b_\ell^+ \text{ for } \ell \neq k \\ (1-\phi)(1-\psi) & b_k^- = 1,\ b_k^+ = 0 \text{ and } b_\ell^- = b_\ell^+ \text{ for } \ell \neq k \\ (1-\phi)\psi + \phi & b_k^- = 1,\ b_k^+ = 1 \text{ and } b_\ell^- = b_\ell^+ \text{ for } \ell \neq k \\ 0 & \text{otherwise} \end{cases} \tag{5}$$

The first option (i.e., the first line on the right-hand side of **Equation 4** and **Equation 5**) describes the probability of transition between an initial state with patient k exiting uncolonized ($b_k^- = 0$) and a final state in which the new patient remains uncolonized ($b_k^+ = 0$) despite possible prior-to-new patient colonization, as shown in **Fig 1a in Main Text**. The other, non-exiting patients in the room do not change colonization status during an exit/entry event ($b_\ell^- = b_\ell^+$ for $\ell \neq k$). If the entering patient is uncolonized ($S = 0$), then in the final state that patient will certainly (with probability 1) remain uncolonized because the prior patient was uncolonized. If the entering patient is colonized $S = 1$, the probability of

the k-th patient being uncolonized is 0. If the status of the entering patient is unknown (**Fig 1b in Main Text**), the probability is $1 - \phi$.

The second option (the second line on the right-hand side of **Equation 4** and **Equation 5**) describes the probability of transitioning from an initial state in which the exiting patient is uncolonized ($b_k^- = 0$) to a final state in which the new patient is colonized ($b_k^+ = 1$). If the new patient is colonized ($S = 1$), the probability that the new patient will still be colonized after entry into the unit is, of course, equal to 1 (**Fig 1c in Main Text**). If the new patient is uncolonized ($S = 0$), it is impossible to transition to a final state in which the k-th patient is colonized (**Fig 1a in Main Text**). If the new patient has an unknown status, then the probability that the k-th patient will be colonized in the final state is ϕ, the pre-existing colonization probability (**Fig 1b in Main Text**).

The third option (the third line on the right-hand side of **Equation 4** and **Equation 5**) describes the transition from a state in which the exiting patient is colonized ($b_k^- = 1$) to a final state in which the new patient is uncolonized ($b_k^+ = 0$) but the other patients remain in the same colonization status (**Fig 1d in Main Text**). This transition can only occur if the new patient enters uncolonized ($S = 0$ if the colonization status is known, else with a probability of being uncolonized of $1 - \phi$) and subsequently no prior-to-new-patient occurs (probability $1 - \psi$).

Finally, the fourth option (the third line on the right-hand side of **Equation 4** and **Equation 5**) describes the transition probability of exit/entry event in which the exiting patient is colonized ($b_k^- = 1$) and the entering patient is uncolonized ($S = 0$ if the colonization status is known, probability $1 - \phi$ if unknown) but becomes colonized ($b_k^+ = 1$) because of prior-to-new patient transmission (**Fig 1e in Main Text**).

During the transition, the other patients remain in the same state. Each of the first four options (the nonzero probabilities) is repeated $2^{n-1}n$ times in an n-bed unit, since the situation it envisions could happen in any one of n beds, and then there are 2^{n-1} states of the remaining beds that do not affect the probability of the transition in question. The matrix as a whole is 2^n by 2^n, but most of its entries are zero.

For convenience, in the main body of the paper, we will drop the arguments and designate the specific matrix $M(S; k, \phi, \psi)$ that corresponds with a replacement event at time t_z as $M(z)$.

Of note, inference of ϕ involves only patients with unknown incoming colonization statuses. If in fact all patients' incoming colonization statuses were known, ϕ would no longer play a role in model inference as it could be directly calculated. If there were relatively few patients with unknown incoming colonization statuses compared to those with known statuses, then the value of ϕ might be unreliable because it was inferred from relatively few data points compared to the estimate of ϕ derived from the colonized fraction of tested patients. In our dataset, very few patients have known pre-existing colonization statuses, so the difference is likely more theoretical than practical in terms of changing the parameter estimates. Future work could include merging estimates of pre-existing colonization derived from incoming patients with both known

and unknown colonization status.

Appendix S1-G

Construction of the Continuous-Time Transition Rate Matrix

In this appendix, we outline a method to construct the continuous-time transition rate matrix for non-turnover intervals. Consider the time interval between t_z^+ and t_{z+1}^- during which no patient replacements occur within a hospital unit (**Fig 3 in Main Text**). We wish to determine the overall probability matrix P for transition from state $\mathbf{b}(t_z^+)$ to $\mathbf{b}(t_{z+1}^-)$ over this time interval. To do so, we use an instantaneous *rate* matrix R, which governs transitions involving a single event that occurs over an infinitesimal time step dt. Without patient replacement, only patient–patient transmission can change the state of the hospital unit by increasing the number of colonized patients within the room (if at least one patient is already colonized). Even though only a single event can occur at a time, multiple transmissions may occur during the time interval between t_z^+ and t_{z+1}^-, so the final state of the hospital unit may have more than one additional colonized patients than the initial state.

Let $|\mathbf{b}| = \sum_{k=1}^{n} b_k$ be the number of colonized patients in a unit. Because the time interval under consideration does not include any patient replacement events, the number of colonized patients can only increase with time. Thus, $|\mathbf{b}(t_z^+)| \leq |\mathbf{b}(t_{z+1}^-)|$. Furthermore, patients colonized in the initial state $\mathbf{b}(t_z^+)$ must also be colonized in the final state $\mathbf{b}(t_{z+1}^-)$. Let us define the relation $\subset$ for any two hospital unit states $\mathbf{a}$ and $\mathbf{b}$ as follows:

$$\mathbf{a} \subset \mathbf{b} := \{k : a_k = 1\} \subset \{k : b_k = 1\} \tag{6}$$

This relation means that all of the colonized beds in $\mathbf{b}$ are also colonized in $\mathbf{a}$. Thus, $\mathbf{b}(t_z^+) \subset \mathbf{b}(t_{z+1}^-)$ because the patients colonized in the initial state must also be colonized in the final state, given that no patient replacement occurred between. Notice that $\mathbf{a} \subset \mathbf{b}$ implies that $|\mathbf{a}| \leq |\mathbf{b}|$, but the converse is not true: $|\mathbf{a}| \leq |\mathbf{b}|$ does *not* imply that $\mathbf{a} \subset \mathbf{b}$. For example, for a 4-bed room, $0001 \not\subseteq 1110$ even though $|0001| < |1110|$. Thus, even though there are fewer colonized patients in the state 0001 than in the state 1110, it is not possible for 0001 to be the initial state of a non-turnover interval for which 1110 is the final state. This is because of our assumption that any patient who has become colonized remains colonized for the duration of that patient's stay in the unit.

Finding the transition rate matrix R The probability per unit time of an *instantaneous* state change from state $\mathbf{a}$ to $\mathbf{b}$ can be described by the transition rate matrix R. Each matrix entry, the transition rate $R_{\mathbf{ab}}$, is governed by the overall patient–patient transmission for the hospital unit and is proportional to the number of colonized patients in the initial state $\mathbf{a}$:

$$R_{\mathbf{ab}} = \begin{cases} |\mathbf{a}|\gamma & \text{if } |\mathbf{b}| = |\mathbf{a}| + 1 \text{ and } \mathbf{a} \subset \mathbf{b} \\ 0 & \text{otherwise} \end{cases} \tag{7}$$

Note that $R_{\mathbf{ab}}$ does *not* contain a factor $n - |\mathbf{a}|$, which is the number of patients available to become colonized in the state $\mathbf{a}$. This is because we are considering each such patient separately. In fact, for a given state $0 < |\mathbf{a}| < n$, the number of $R_{\mathbf{ab}}$ such that $R_{\mathbf{ab}}$ is nonzero is exactly $n - |\mathbf{a}|$. For $|\mathbf{a}| = 0$, the case where no patients are colonized, no transmission can occur, so the only possible final state is the state in which all patients remain uncolonized. Similarly, for $|\mathbf{a}| = n$, all patients are already colonized, so the only possible final state is that in which all patients are colonized.

Individual transitions are only possible between states in which the set of colonized beds in the prior state is a subset of the set of colonized beds in the subsequent state, and in which the number of colonized beds in the prior state is exactly one fewer than in the subsequent state. For example, the transitions from 0001 to 1001, 0101, and 0011 are possible (and each have a rate γ), but the transitions from 0001 to 1100, 1010, and 0110 are not possible, even though the number of patients colonized increases by 1. Recall, however, that there can be more than one individual transition (e.g., a patient-patient transmission) between adjacent exit/entry events. Thus, between any two such events, the number of colonized patients can increase by more than one, but there is still the restriction that the set of colonized beds at the start of such an interval is a subset of the set of beds at the end of the interval.

Appendix S1-H

Converting the Patient–Patient Transmission Matrix from a Continuous-time Rate Matrix to a Discrete-Time Probability Matrix

For an arbitrary time interval in which no exit/entry events occur, it is mathematically convenient to compute the discrete-time probability of patient–patient transmission over the interval from the continuous-time rate (probability per unit time) given an initial and final state of the unit. This useful conversion allows us to consider the states of the hospital unit only at observation times yet take into account all possible states that the hospital unit may have evolved through when unobserved. Calculation of the probability matrices over time intervals of any length allows use of hospital surveillance data observed at any time, but it requires calculation of a matrix exponential for every time interval of a different length.

In this appendix, we convert the continuous-time, discrete-state matrix R (which describes patient–patient transmission rates between exit/entry and/or test events) to a discrete-time, discrete-state probability matrix $P(z)$ describing the transition between an initial state at time t_z and final state at time t_{z+1}. To do so, we transform the *rate* matrix R over time intervals $t_z - t_{z-1}$ (for $z \in \{1, 2, \dots\}$) into a discrete-time, discrete-state *probability* matrix $P(z)$ that describes the probability of transitioning from the state vector $\mathbf{b}(t_{z-1}^{+})$ to $\mathbf{b}(t_z^{-})$ by solving a matrix differential equation[4] for a particular time interval.

Notice that the probability of transitioning between states from times t_z^{+} to t_{z+1}^{-} is equal to the probability of transitioning between those same states for any time interval of the same length, such as from 0 to $t = t_{z+1}^{-} - t_z^{+}$. For convenience,

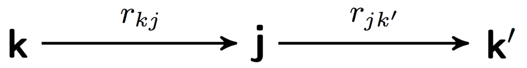

Figure E. Flow of probability into and out of state j. Here, r_{kj} is the probability per unit time of transitioning to state j from state k, and $r_{jk'}$ is the probability per unit time of transitioning to state k' from state j.

we will calculate $P(t)$ with $t = t^-_{z+1} - t^+_z$ for all non-turnover intervals. However, we could calculate the transition probability matrix for any length of time in which no patient replacement occurs.

Although at any instant only one bed can change state, over time, multiple events can occur. Thus, the instantaneous *rate* matrix will be sparse, but the *probability* matrix for the transition between initial state i to j at time t will be less sparse (but not full because certain transitions are impossible in our model). Here, i and j are matrix indices that correspond to state vectors in any arbitrarily chosen way. We find the matrix of transition probabilities by solving the differential equation

$$\frac{dP_{ij}(t)}{dt} = \sum_{k=1}^{k_{max}} P_{ik}(t) R_{kj} - \sum_{k'=1}^{k_{max}} P_{ij}(t) R_{jk'} \tag{8}$$

on the interval $(0, t)$. Note that this is essentially the forward Kolmogorov equation[10, 12]. The first term on the right side of the equation refers to the probability per unit time of all ways of entering state j at time t given that the system was in the initial state i at $t = 0$. The second term refers to the probability per unit time of all ways of leaving state j at time t, given that the system was initially in state i. (The transitions into and out of state j are shown in **Fig E**.)

The right side can be written as a single sum

$$\frac{dP_{ij}}{dt} = \sum_{k=1}^{k_{max}} P_{ik} A_{kj} \tag{9}$$

where

$$A_{kj} = \begin{cases} R_{kj} & k \neq j \\ -\sum_{k'=0}^{k_{max}} R_{jk'} + R_{jj} & k = j \end{cases} \tag{10}$$

Here, R_{jj} refers to diagonal elements R_{jk} where $j = k$, or possible "transitions" in which the final state and initial state are the same. Note that any diagonal elements R_{jj} refer to processes do not change state, so they cancel in evaluation of A and have no effect in $P_{ij}(t)$. Thus, A_{jj} may alternatively be written

$$A_{jj} = -\sum_{k'=0, k' \neq j}^{k_{max}} R_{jk'} \tag{11}$$

The diagonal elements of A are the negative sum of their row's off-diagonal elements. In terms of A, P satisfies the matrix differential equation

$$\frac{dP(t)}{dt} = P(t)A \tag{12}$$

At $t = 0$, with no time elapsed, the unit will remain in the same state, so $P_{ii}(0) = 1$. Other final states for an interval of zero duration have zero probability, since there is no time for anything to happen, so $P_{ij}(0) = 0$ for $i \neq j$. The initial condition is therefore $P(0) = I$, where I is the identity matrix. With this initial condition, the solution to the matrix differential equation at time t is

$$P(t) = e^{At} \tag{13}$$

The matrix exponential can be written as a power series[4] in which the matrix exponential is defined as

$$e^{AT} = \sum_{n=0}^{\infty} \frac{(AT)^n}{n!} = I + At + \frac{1}{2!}(At)^2 + \cdots \tag{14}$$

This gives the discrete-time probability matrix of transitioning to state j after time $t = T$ given that the unit was in state i at $t = 0$. In MATLAB, the matrix exponential can be calculated using the function **expm**, but we present an alternate method using Jordan form in **Appendix S1-O**. This alternative method can be used to reduce the time needed to compute the maximum-likelihood parameters.

Appendix S1-I

Parameter Search Plots

Fig F shows the likelihood contour plot of a parameter search for ϕ versus γ at different values of ψ. The color bar at the right indicates the log likelihood value of the parameter set.

Appendix S2-J

Discrete Evolution of the Probability Distribution

Fig 4 in Main Text gives a simple example of (**a**) constructing a time line for a sequence of exit/entry and test events based on (**b**) a surveillance test scheme for a hospital unit. The timeline is divided into **non-turnover intervals** of length $t^-_{z+1} - t^+_z$ by tests and exit/entry events. (Unlike exit/entry events, whose state changes instantaneously from t^-_z to t^+_z, a test event has only one state at t_z, so $t_z = t^-_z = t^+_z$.) Each non-turnover interval corresponds to a matrix $P(z)$, but the instantaneous transition of an exit/entry event at time t_z corresponds to probability matrix $M(z)$. After construction of P matrices for all non-turnover intervals, we will choose sub-matrices of $P(z)$ and $M(z)$ whose dimensions are dictated by the possible states consistent with observations at the initial and

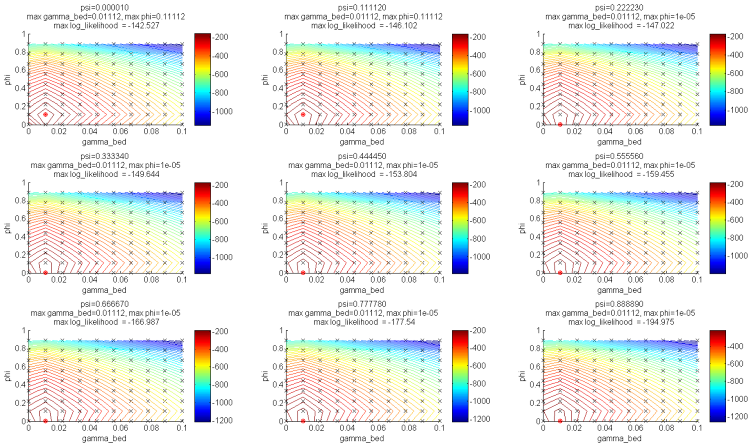

Figure F. Contour plot for parameter search for ϕ versus γ at different values of ψ. The color bar at the right indicates the log likelihood value of the parameter set.

final times. Finally, we take the product of the initial probability distribution p_0 and the $P(z)$ and $M(z)$ sub-matrices (in order of event occurrence) to calculate the overall probability of observations given the model.

Let the row vector p be the probability distribution over possible states of the unit. The total number of possible states is discrete, finite, and increases exponentially with the number of beds in the room (2^n). Each element p_i describes the probability of being in state i at time t_z. The probability distribution p evolves in discrete steps at patient turnover events, and continuously between such events. If, however, we choose to do so, we can think of the system as if it jumps from one patient turnover event to the next, and then we have a fully discrete evolution in which patient-turnover events alternate with the changes in p that occur over non-turnover intervals.

We assume that the unit starts with all patients uncolonized, so p_0 is a row vector of with 2^n elements, all but one that are equal to zero. The element that is equal to 1 is the one corresponding to the state with all patients uncolonized. The probability distribution at time t_Z, given an initial probability distribution p_0, turnover events at times $\{t_1, t_2, \ldots, t_{Z-1}\}$, and final time t_Z is

$$p = p_0 P(0) M(1) P(1) \ldots M(Z-1) P(Z-1) \tag{15}$$

Here, $P(0)$ is the transition probability matrix associated with patient–patient transmission prior to the first patient turnover event, M(1) is the transition probability matrix associated with the first patient turnover event, P(1) is the transition probability matrix associated with the non-turnover interval following the first patient turnover event, and so on.

Note that the times at which tests occur are treated exactly like patient

turnover times, except that no patient turnover occurs, so the corresponding M matrix is the identity.

Observation Model We have now described the evolution of the probability distribution with time of states of the hospital unit. In the next sections, we incorporate observations into this model. Consider a series of exit/entry or observation events that occur at times $t_1, t_2, \ldots t_{Z-1}$. In the next section, we will determine the probability distribution of the state of the unit at time t_Z after all observations and replacement events.

We assume (1) test events occur only between and not during patient replacement events; (2) tests are accurate; and (3) once positive, patients do not become decolonized during their stay. Consequently, if a patient tests positive, we assume all subsequent tests are positive until exit. If a patient tests negative, we assume the status at prior tests or events was negative from the time of entry. (This method is similar to the Cooper et al. method of data augmentation[3].) We use this extrapolated data for all subsequent sections, so any reference to test results in following sections will refer to the extrapolated test results.

Probability of a sequence of states Consider a sequence of states and their corresponding matrices that occur at given times t_z. We will renumber all these lists in temporal order with integer indices j so that there are no longer limits (such as z^- or z^+). We take a list of times (such as $t_0, t_1^-, t_1^+, t_2^-, \ldots, t_Z$ from the system shown in **Fig 4 in Main Text**) and re-number them with integer indices j in the following manner: $\tau_0 = t_0$, $\tau_1 = t_1^-$, $\tau_2 = t_1^+$, $\tau_3 = t_2^-, \ldots, \tau_\zeta = t_Z$. Similarly, we create a list of matrices $W(j)$ whose indices are integers that correspond to times τ_j. (For example, the corresponding list would be $W(0) = P(0)$, $W(1) = M(1)$, $W(2) = P(1)$, $W(3) = P(2)$, ..., $W(\zeta) = P(Z)$).

Last, we convert the series of states $\mathbf{b}(z)$ to a linear list $\mathbf{a}(j)$ indexed by the integer j. (For example, $\mathbf{a}(1) = \mathbf{b}(1)^-$, $\mathbf{a}(2) = \mathbf{b}(1)^+$, and so on).

Now the probability of any given sequence of states at times $\tau_0, \tau_1, \ldots, \tau_\zeta$ is

$$\begin{aligned}\Pr(X(0) = \mathbf{a}(0) \text{ and } X(1) = \mathbf{a}(1) \text{ and } \ldots \text{ and } X(\zeta) = \mathbf{a}(\zeta)) \\ = p_0(\mathbf{a}(0))\; W(0)_{\mathbf{a}(0),\mathbf{a}(1)}\; W(1)_{\mathbf{a}(1),\mathbf{a}(2)}\; \ldots\; W(\zeta-1)_{\mathbf{a}(\zeta-1),\mathbf{a}(\zeta)}\end{aligned}$$

Here, p_0 is the initial probability distribution, and $p_0(\mathbf{a}(0))$ is the initial probability of being in state $\mathbf{a}(0)$. $\mathbf{a}(i)\mathbf{a}(j)$ is the probability of transitioning from state $\mathbf{a}(i)$ at time τ_i to state $\mathbf{a}(j)$ at time τ_j.

Probability of sets of states consistent with observations We have determined the probability of a given sequence of states for a system. However, during an observation event, only a subset of patients may be tested, so we may only have partial information about the state of the hospital unit. If not all beds are tested, there will be multiple possible states consistent with observations. For example, consider the first set of tests at time t_2 in **Fig 4 in Main Text** . If we assume all test results (for the white open circles shown) are negative, then

the partially observed state of the unit is 00?0. The two possible underlying states consistent with test results are $\{0000, 0010\}$.

Let $B(j)$ be the set of all possible states consistent with test results at time τ_j. Each state vector $\mathbf{b}(j)$ in the set $B(j)$ will have some components b_k (for k in the set of tested beds) whose status is known from test results, but the remaining components can take on any combination of values (0 or 1). Notice that if all tests are performed, the state of the system will be known and $B(j)$ will have only one element. If no tests are performed, all states are possible, so $B(j)$ will have 2^n elements. Thus, every observation with m tests performed (for $m \in \{0, 1, \ldots, n\}$) yields a set of 2^{n-m} possible states consistent with its test results.

The true state of the system $X(j)$ at time τ_j is contained in the set of possible states $B(j)$. Therefore, the whole set of observations gives us the following information about the series of true system states at all of the test times:

$$[X(0) \in B(0)] \text{ and } [X(1) \in B(1)] \text{ and } \ldots \text{ and } [X(\zeta) \in B(\zeta)] \tag{16}$$

This can be written in matrix notation (and evaluated that way in MATLAB) if we introduce the following subvector and submatrices. Let $p_0(B(0))$ be the row vector with entries of initial probabilities for each of the possible initial states in set $B(0)$. Let $W(j, B(j), B(j+1))$ be the rectangular submatrix of $W(j)$ containing the rows corresponding with the decimal state indices specified in $B(j)$ and the columns specified by $B(j+1)$. Finally, let $U(B(\zeta))$ be a vector with all elements equal to 1 and the same number of elements as contained in $B(\zeta)$. Then the overall probability is

$$\begin{aligned}\mathcal{P} &= p_0(\mathbf{a}(0))\ W(0, B(0), B(1))\ W(1, B(1), B(2))\ \ldots\ W(\zeta-1, B(\zeta-1), B(\zeta))\, U(B(\zeta)) \\ &= p(\mathbf{a}(0)) \prod_{j=0}^{\zeta-1} W(j, B(j), B(j+1))\end{aligned}$$

Because matrix multiplication is not commutative, we assume in the above product notation that the matrices $W(j, B(j), B(j+1))$ are multiplied from left to right in order of index j. To find the parameters for prior-to-new patient probability ψ and transmission rate γ, we must maximize the likelihood $\mathcal{P}$ with respect to a particular set of observations: patient exit/entry events, the corresponding new patient colonization status, and results of testing particular beds at given times.

Appendix S1-K

Reduced Model and Inference Method In this appendix, we outline the reduced model and the associated inference method. The reduced model is essentially the classic continuous-time birth and death process[1] applied to a susceptible-infective-susceptible (SIS) model. In contrast to the full detailed model, which tracks the state of each individual patient, we define the state of

the reduced model $i \in \{0, 1, \dots n\}$ as the number of colonized patients in the unit. The number of colonized patients can increase from state i to $i+1$ with rate f_i for $i \in \{0 \dots n-1\}$, and the number of colonized patients can decrease from $i+1$ to i with rate g_{i+1} for $i \in \{1 \dots n\}$.

Rate of increase The rate of transition from state i to $i+1$ (for $i \in \{0, 1, \dots, n-1\}$) depends on the patient-patient transmission rate and the probability of replacement of an uncolonized patient by colonized patient:

$$f_i = \gamma i(n-i) + \beta(n-i)\phi \tag{17}$$

The rate of increase caused by patient-patient transmission depends on the number of colonized-uncolonized pairs of patients $i(n-i)$ and γ, the patient-patient transmission rate per colonized patient per day. The rate of increase caused by the replacement of an uncolonized patient by a colonized patient depends on the turnover rate per day (β), the probability of pre-existing colonization (ϕ), and the number of uncolonized patients ($n-i$) that can be replaced. When all patients in the unit are colonized ($i=n$), it is not possible to increase the number of colonized patients, so $f_n = 0$. The processes of patient-patient transmission and replacement of an uncolonized patient by a colonized patient increase the number of colonized patients by one. However, no term appears for prior-to-new patient colonization because replacement of a colonized patient by a patient who becomes colonized does not change the net number of colonized patients.

Rate of decrease The overall rate of decrease from state i to state $i-1$ (for $i \in \{1 \dots n\}$) depends on processes that decrease the number of colonized patients by one. In this model, the only way to decrease i is to replace a colonized patient by an uncolonized patient who remains uncolonized (no prior-to-new patient colonization):

$$g_i = \beta i(1-\phi)(1-\psi) \tag{18}$$

However, when $i = 0$, there are no colonized patients left to exit, so $g_0 = 0$.

Differential equations Let P_i be the probability of being in state i. As time passes, the probability of being in a particular state will change. For the state ($i=0$), which has no patients colonized, the differential equation for the change in P_i is

$$\frac{dP_0}{dt} = g_1 P_1 - f_0 P_0 \tag{19}$$

For an intermediate state $i \in \{1, 2, \dots, n-1\}$, the differential equation is

$$\frac{dP_i}{dt} = f_{i-1}P_{i-1} + g_{i+1}P_{i+1} - f_i P_i - g_i P_i \tag{20}$$

Finally, for the state $i = n$ in which all patients are colonized, the differential equation is

$$\frac{dP_n}{dt} = f_{n-1}P_{n-1} - g_n P_n \tag{21}$$

For the sake of simplicity of analysis, we assume that the hospital unit has reached steady state in which the overall rates of entering and exiting the state are equal. At steady state, there is no change in the probability of being in any particular state, so the time derivatives of P_i can be set equal to zero. If we solve **Equation 19** for P_1, we find that $P_1 = f_0 P_0 / g_1$. By induction (described below), we find the equations for $i \in \{2, \ldots, n\}$:

$$P_i = \frac{f_{i-1}}{g_i} P_{i-1} \tag{22}$$

This equation shows the probability for the next higher state P_{i+1} in terms of the present state P_i. Substituting in recursively and solving for the probability P_i in terms of P_0, for $i \in \{1, 2, \ldots, n\}$, we find

$$P_i = \prod_{j=0}^{i-1} \frac{f_j}{g_{j+1}} P_0 \tag{23}$$

The sum of probabilities over all possible states is 1, so $P_0 + \sum_{i=1}^{n} P_i = 1$. Substitute **Equation 23** for P_i:

$$P_0 + \sum_{i=1}^{n} \left(\prod_{j=0}^{i-1} \frac{f_j}{g_{j+1}} \right) P_0 = 1 \tag{24}$$

We then solve for P_0:

$$P_0 = \frac{1}{1 + \sum_{i=1}^{n} \left(\prod_{j=0}^{i-1} \frac{f_j}{g_{j+1}} \right)} \tag{25}$$

The equations for P_i and P_0 are general and hold regardless of the actual rates f_j or g_j.

Proof by induction The base case for $i = 0$ is as follows: We set **Equation 19** equal to zero. Then we can rearrange the equation to obtain $g_1 P_1 = f_0 P_0$. For the inductive step, we show that if the equations for i hold, then the equations for $i + 1$ hold. In other words, we demonstrate that if $g_i P_i = f_{i-1} P_{i-1}$ holds, then $g_{i+1} P_{i+1} = f_i P_i$ also holds. First, we substitute the former equation into the steady state version of **Equation 20**, with the result:

$$g_{i+1} P_{i+1} - f_i P_i = 0$$

Then, by rearranging this equation, we see that indeed, $g_{i+1} P_{i+1} = f_i P_i$. Since both the base case and inductive step have been performed, the statement $g_{i+1} P_{i+1} = f_i P_i$ holds for $i = 0, \ldots, n - 1$, as claimed. (Although we did not make any use of **Equation 21** here, note that the case $i = n - 1$ of the foregoing is the same as the steady-state version of **Equation 21**. This comes about because the sum of the probabilities is equal to 1, so any one of the equations is implied by all of the others.)

Reduced Model Inference Method Consider a series of N observations made at times $t_0, t_1, \ldots, t_N$. Let the state at time t_z be an integer i_z that gives the number of colonized patients within the unit. Let W be a matrix in which each entry $W_{i,j}$ describes the probability of transitioning between state i and state j. The matrix W is constructed in a manner analogous to that shown in **Appendix S1-H** except that the continuous-time, discrete-state rate matrix R is composed of entries $r_{i,j}$ that are the *overall* rates of transition between states i and j for the continuous-time reduced model from all mechanisms: pre-existing colonization during patient turnover, bed-to-patient transmission, and patient-patient transmission.) In the case of the reduced model, $r_{i,i+1} = f_i$ for $i \in \{0 \ldots n-1\}$) and $r_{i,i-1} = g_i$ for $i \in \{1 \ldots n\}$).

The probability of any given sequence of states at the observation times $0, t, \ldots, t_N$ is

$$Pr(X^0 = i_0 \text{ and } X^1 = i_1 \text{ and } \ldots \text{ and } X^N = i_N) = p_{i_0} W^0_{i_0 i_1} W^1_{i_1 i_2} \ldots W^{N-1}_{i_{N-1} i_N} \tag{26}$$

However, the state of the system may not fully observed at the observation times. Instead, a subset of patients are tested to see whether they have the infection or not. At an observation time, suppose that s patients are tested and i' are found to be colonized. Then $0 \leq i' \leq s \leq n$, and we know that the actual number of patients colonized (i) satisfies $i' \leq i \leq i' + (n-s)$. Here, the lower bound for the number of colonized patients is simply the number that tested positive for colonization (i'). However, the upper bound is the sum of the number of patients known to be colonized and the untested patients (which could potentially all test positive). Thus, every observation yields an interval of values in which the actual number of infections is known to be.

Let the interval that is found in this way at the time t_j be denoted I^j. Then the whole set of observations gives us the information that the true value of each state is contained somewhere within the interval I^j:

$$(X^0 \in I^0) \,\&\, (X' \in I') \,\&\, \ldots \,\&\, (X^N \in I^N) \tag{27}$$

The likelihood (a priori probability) of this set of observations is given by

$$\mathcal{P} = \Pr((X^0 \in I^0) \,\&\, (X^1 \in I^1) \,\&\, \ldots \,\&\, (X^N \in I^N)) \tag{28}$$

$$= \sum_{i_0 \in I^0} p_{i_0} \sum_{i_i \in I^1} W^0_{i_0 i_1} \sum_{i_2 \in I^2} W^1_{i_1 i_2} \cdots \sum_{i_N \in I^N} W^{N-1}_{i_{N-1} i_N} \tag{29}$$

This can be neatly written in matrix notation (and evaluated that way in MATLAB) if we introduce the following subvector and submatrices. Let $p(I^0)$ be the row vector with the entries of p specified by I^0, and let $W(I^n, I^{n+1})$ be the rectangular submatrix of W containing the rows specified by I^n and the columns specified by I^{n+1}. Finally, we need the vector $U(I^N)$ which has all elements equal to 1 and a number of elements equal to the number of elements in I^N. In terms of this notation, we have

$$\mathcal{P}_{\mathcal{R}} = p(I^0) W(I^0, I^I) W(I^1, I^2) \ldots W(I^{N-1}, I^N) U(I^N) \tag{30}$$

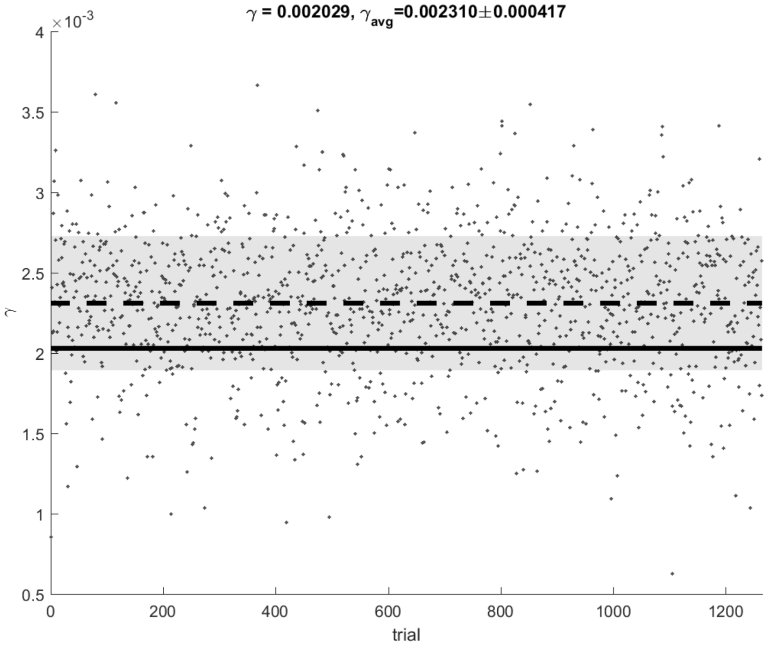

Figure G. Reduced model parameter results of 1265 simulations for γ. The solid black line shows the input parameter, the dashed black line shows the mean best-fit parameter over all simulations, and the gray region shows the standard deviations.

This gives a convenient way in MATLAB to evaluate the likelihood of any particular set of observations. In general, our task is to maximize $\mathcal{P}_R$ with respect to $f_0 \dots f_{n-1}$ and $g_1 \dots g_n$; in this particular case, we maximize with respect to β, γ, ϕ, and ψ as f_i is a function of β, γ, and ϕ, and g_i is a function of β, ϕ, and ψ. It is straightforward to evaluate $\mathcal{P}_R$ for any particular choice of parameters by the method outlined above.

Appendix S1-L

Reduced Model Inference Results

Figures G-I show the results for inference for 1265 sets of simulated data created using the set of best-fit parameters from the full inference method applied to actual hospital data. Notice that for γ and ψ, the input parameter for the simulations falls within the standard deviation error bars of the output mean best-fit parameter for the set of 1265 simulated trials, but for ψ, the mean estimated best-fit parameter is not close to the input parameter.

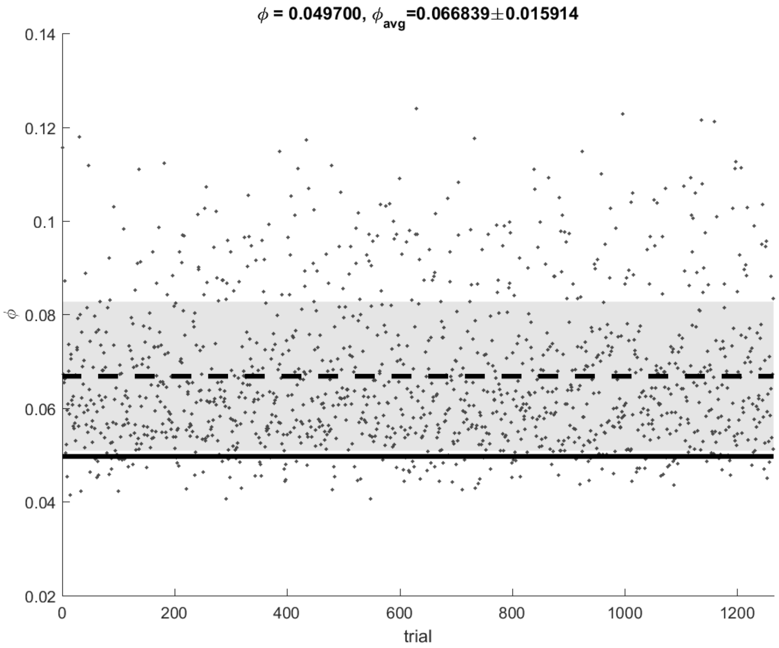

Figure H. Reduced model parameter results of 1265 simulations for ϕ. The solid black line shows the input parameter, the dashed black line shows the mean best-fit parameter over all simulations, and the gray region shows the standard deviations.

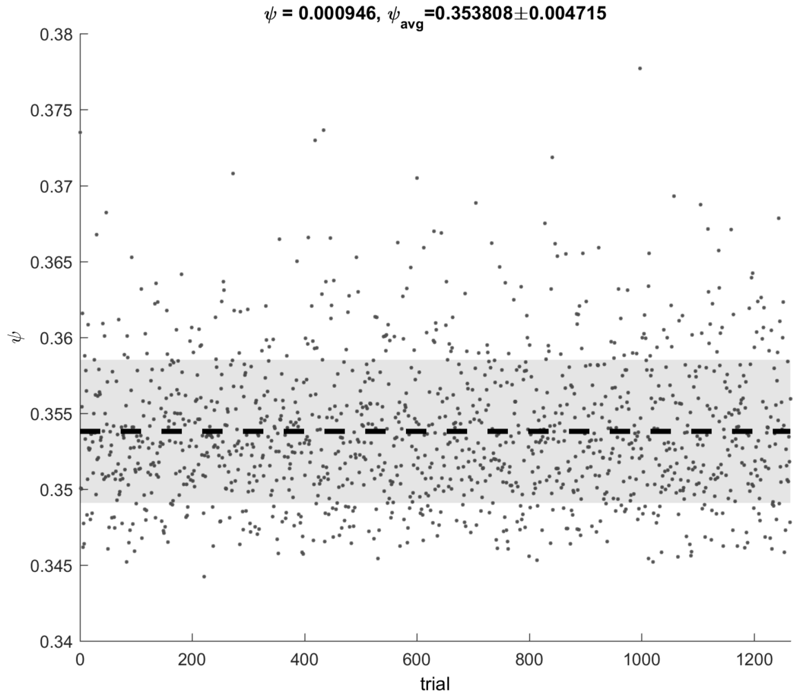

Figure I. Reduced model parameter results of 1265 simulations for ψ. Each data point shows the best-fit parameter result for a particular simulated trial. The solid black line shows the input parameter, the dashed black line shows the mean best-fit parameter over all simulations, and the gray region shows the standard deviations. Note that the parameter value that was actually used in creating these simulations ($\psi = 0.0009$) is off the scale of the plot.

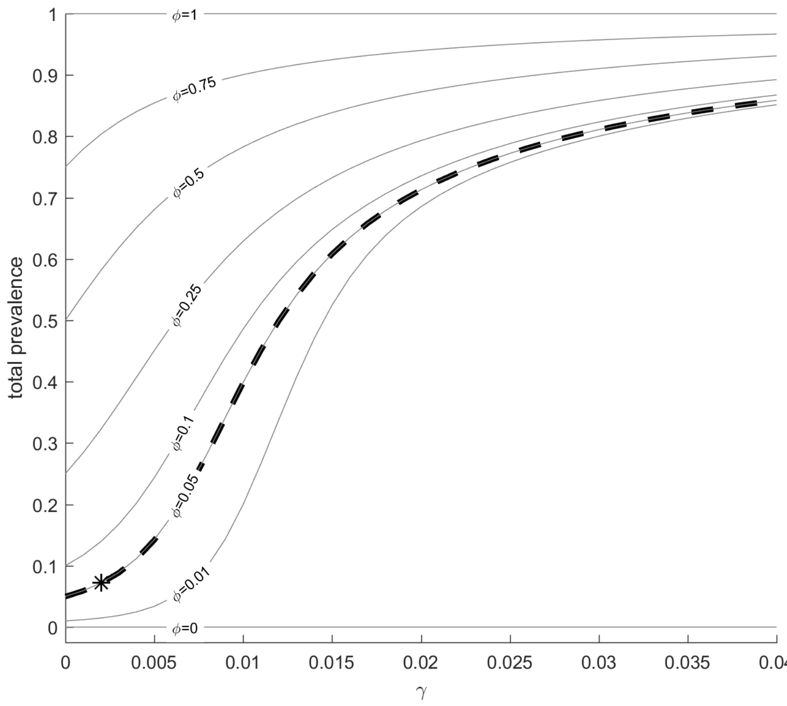

Figure J. Patient–patient transmission rate (per day) versus total prevalence (fraction of patient-days that are colonized) for various values of ϕ. The bold star shows the best-fit patient–patient transmission rate ($\gamma = 0.00203$/day per colonized/uncolonized patient pair), with the dashed line showing the contour at $\phi = 0.0497$/day.

Appendix S1-M

Reduced Model Total Prevalence

Figures J-O show detailed total prevalence plots.

Appendix S1-N

Conversion and "Explosion" of Reduced to Full Matrix

Finding the matrix exponential of the full detailed rate matrix becomes extremely expensive computationally as the number of beds (n) increases because the number of matrix entries increases exponentially (as $(2^n)^2$). However, the size of the reduced state rate matrix A increases only quadratically with the number of beds (as $(n+1)^2$). As it turns out, because of the assumption of linear increase in γ with the number of colonized patients in the unit (for the full model) or the number of pairs of colonized/uncolonized patients in the

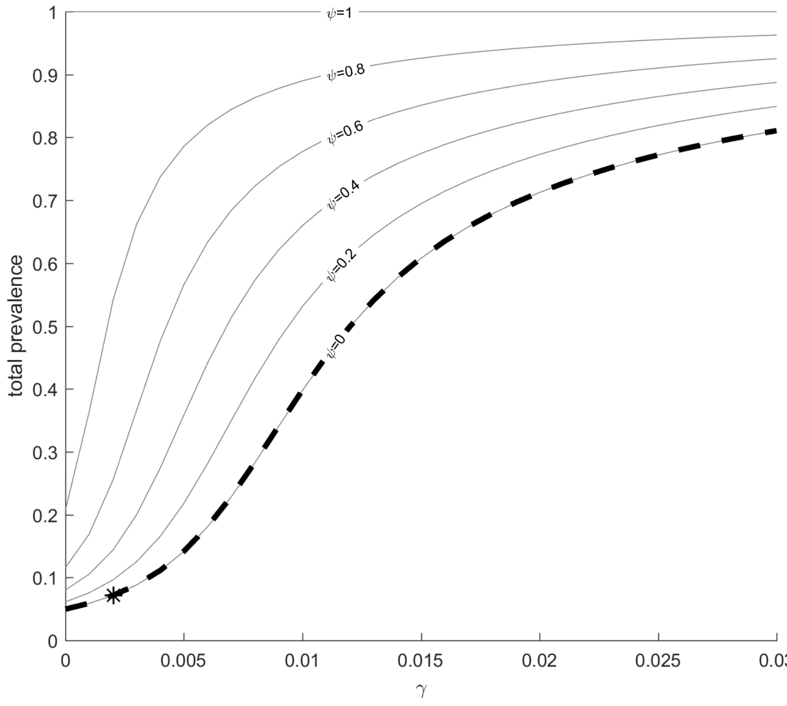

Figure K. Patient–patient transmission rate (per day) versus total prevalence (fraction of patient-days that are colonized) for various values of ψ. The bold star shows the best-fit patient–patient transmission rate ($\gamma = 0.00203$/day per colonized/uncolonized patient pair) with the dashed line showing the contour at $\psi = 0.000946$.

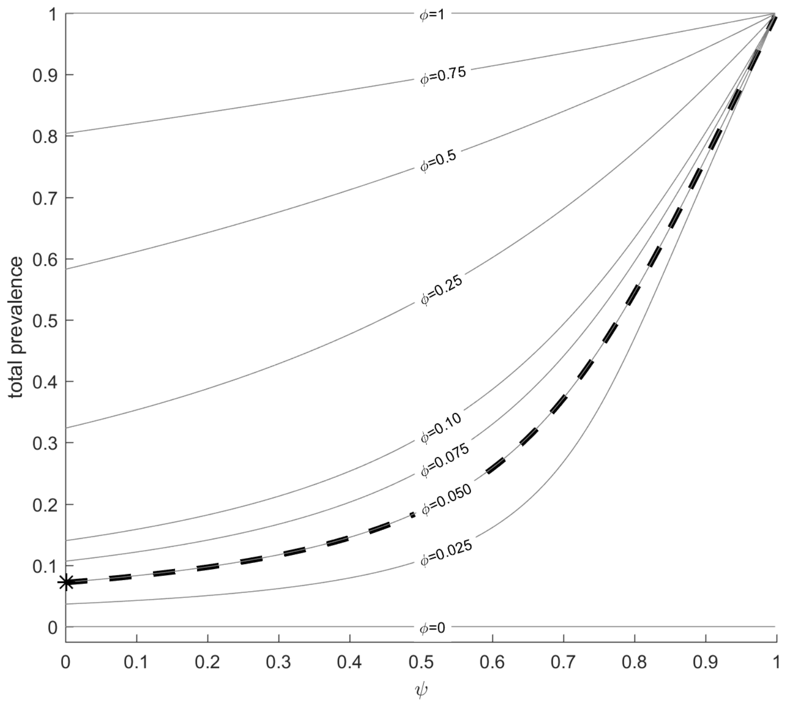

Figure L. Prior-to-new patient transmission probability versus total prevalence (fraction of patient-days that are colonized) for various values of ϕ. The bold star shows the best-fit prior-to-new patient probability ($\psi = 0.000946$), with the dashed line showing the contour corresponding to $\phi = 0.0497$.

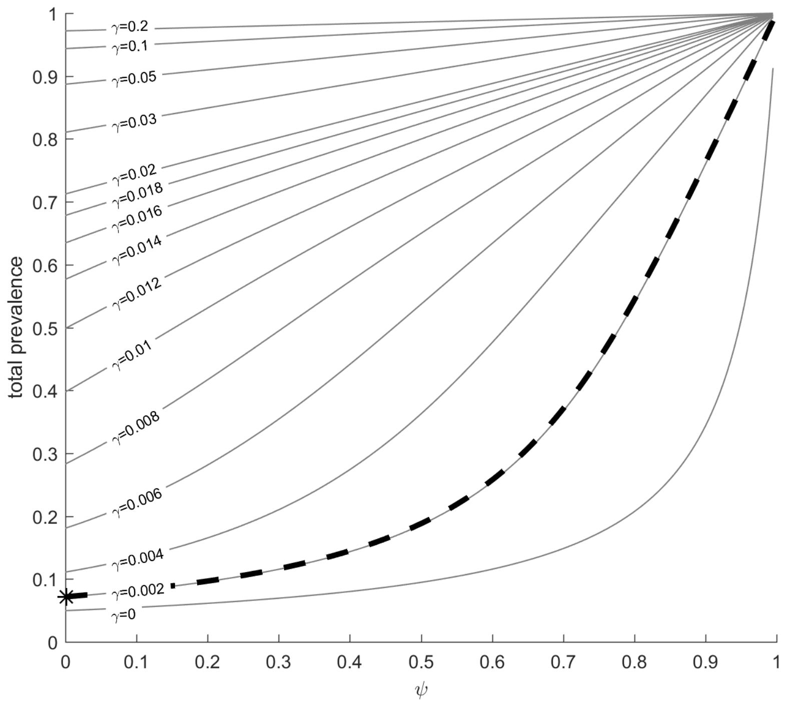

Figure M. Prior-to-new patient transmission probability versus total prevalence (fraction of patient-days that are colonized) for various values of γ. The bold star shows the best-fit prior-to-new patient probability ($\psi = 0.000946$), with the dashed line showing the contour corresponding to $\gamma = 0.00203$.

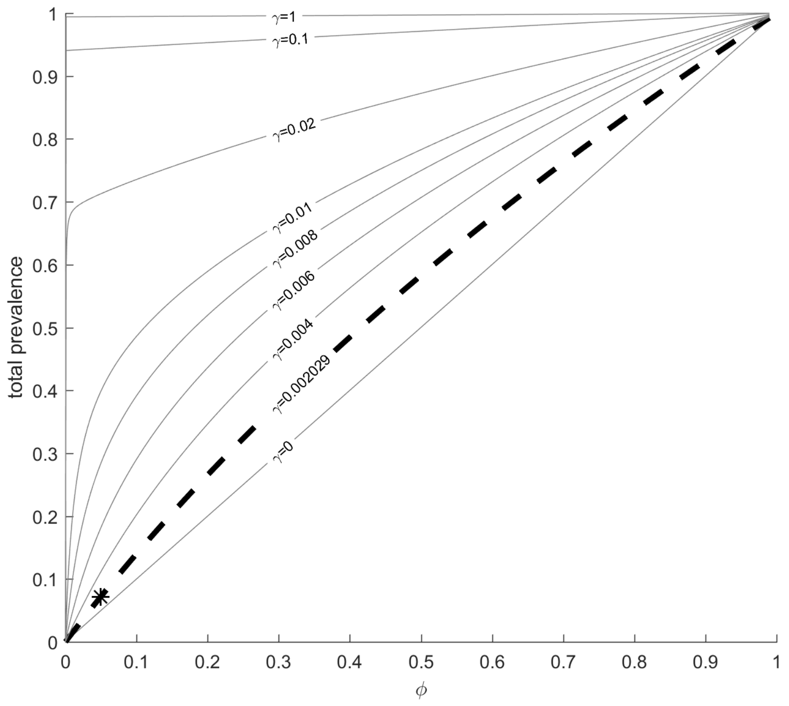

Figure N. Pre-existing colonization probability versus total prevalence (fraction of patient-days that are colonized) for various values of γ. The bold star shows the best-fit pre-existing colonization probability ($\phi = 0.0497$), with the dashed line showing the contour corresponding to $\gamma = 0.00203$.

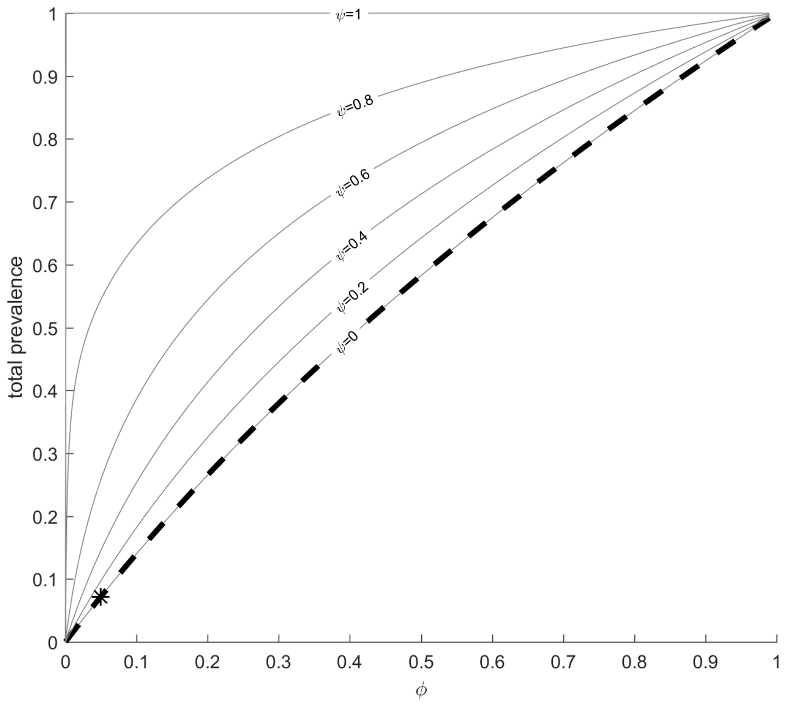

Figure O. Pre-existing colonization probability versus total prevalence (fraction of patient-days that are colonized) for various values of ψ. The bold star shows the best-fit pre-existing colonization probability ($\phi = 0.0497$), with the dashed line showing the contour corresponding to $\psi = 0.000946$.

unit (reduced model), the reduced rate matrix entries can be converted to their equivalents in the full detailed matrix. These entries will be repeated multiple times because each reduced state corresponds to multiple full detailed states. For example, there are four ways that a 4-bed unit can have a single colonized patient (0001, 0010, 0100, 1000). Thus, the reduced matrix entries must be not only converted to their full matrix equivalents but also "exploded" into the correct locations in the full detailed rate matrix Q. (This is essentially an application of a graph-automorphism "lumping" approach[13] to reduce the number of states, albeit limited to just the continuous-time portions of the hybrid model.)

Thus, we can find the matrix exponential for an n-bed room using an $(n+1)$ by $(n+1)$ matrix, then use this method to convert and "explode" its entries to find the matrix exponential of the full detailed matrix. This is the single most important factor in reducing the time needed to calculate the discrete-time probability matrices P from the continuous-time rate matrix. However, for hospital units of size $n = 16$ or less, it can be used in conjunction with the Jordan normal form method outlined in **Appendix S1-O**.

Reduced Rate Matrix First, we find the reduced rate matrix entries in terms of the parameter γ; v, the number of colonized patients, which is also the initial state; and w, the number of colonized patients in the final state. Let $\hat{A}$ be the *reduced* modified rate matrix constructed from the full transmission transition rate matrix R. $\hat{A}$ is an $(n+1) \times (n+1)$ matrix with rows and columns numbered from $0 \ldots n$. The elements of $\hat{A}$ are given by

$$\hat{A}_{vw} = \begin{cases} -\gamma v(n-v) & w = v, v = 0..n \\ \gamma v(n-v) & w = v+1, v = 0 \ldots n-1 \\ 0 & \text{otherwise} \end{cases} \tag{31}$$

This matrix is upper triangular and bidiagonal with eigenvalues given by the diagonal elements $\lambda_v = -\gamma v(n-v)$. Note that the eigenvalues are repeated with multiplicity two, since $\lambda_v = \lambda_{n-v}$. (An exception, if n is even, is $\lambda_{n/2}$, which has multiplicity one.)

Conversion of Rate Matrix to Probability Matrix Next, we convert the continuous-time reduced rate matrix to the discrete-time probability matrix over a given time interval of length t as described in **Appendix S1-H**. Compression of the matrix allows us to perform matrix exponentiation on a matrix that is $(n+1) \times (n+1)$ instead of $2^n \times 2^n$. This is key to reducing the time needed to calculate the likelihood shown in **Equation 2 in Main Text**:

$$\mathcal{P} = p_0\big(\mathbf{a}(0)\big)\; W\big(0, B(0), B(1)\big)\; W\big(1, B(1), B(2)\big) \ldots W\big(\zeta{-}1, B(\zeta{-}1), B(\zeta)\big)\; U\big(B(\zeta)\big)$$

We must then "explode" the matrix back to the equivalent full detailed state probability matrix using the method in the next section. Because the exit/entry matrices are constructed using the full-detailed state, we cannot also compress these matrices and calculate everything in terms of the reduced state

as in **Equation 30**. It is the use of the full-detailed state for exit/entry that allows us to distinguish pre-existing colonization probability ϕ and prior-to-new patient colonization ψ, but the compressed matrix is sufficient for inference of the parameter γ for patient–patient transmission, which depends on the number of colonized patients in the unit.

Full Rate Matrix We find the full detailed state *probability* matrix entries in terms of the reduced state matrix entries. Let $Q_{\mathbf{ab}}(t)$ be the probability that the state of the room is $\mathbf{b}$ at time t, given that the state of the room was $\mathbf{a}$ at time 0. Now $Q_{\mathbf{ab}}(t) = 0$ unless the colonized beds present in state $\mathbf{a}$ are also present in $\mathbf{b}$ (so $\mathbf{a} \subset \mathbf{b}$, as defined in **Equation 6**), since we assume that patients do not spontaneously become decolonized. Let $j = |\mathbf{a}|$ and $k = |\mathbf{b}|$.

If $\mathbf{a} \subset \mathbf{b}$, then $j \leq k$. For any given state $\mathbf{a}$ with $|\mathbf{a}| = j$, the number of possible states $\mathbf{b}$ with $\mathbf{a} \subset \mathbf{b}$ and $|\mathbf{b}| = k$ is

$$\binom{n-j}{k-j} \tag{32}$$

since in the state $\mathbf{a}$, there were $(n - j)$ uncolonized patients, and $k - j$ of these had to become colonized to produce the state $\mathbf{b}$. All of these choices are equally likely, and therefore we have

$$P_{jk}(t) = Q_{\mathbf{ab}}(t)\binom{n-k}{l-k} \tag{33}$$

where $j = |\mathbf{a}|$ and $k = |\mathbf{b}|$. Thus, if the $P_{jk}(t)$ are already known, we can find the entries in the full probability matrix $Q_{\mathbf{ab}}(t)$ in the following way:

$$Q_{\mathbf{ab}}(t) = \begin{cases} \dfrac{P_{|\mathbf{a}||\mathbf{b}|}(t)}{\binom{n-|\mathbf{a}|}{|\mathbf{b}|-|\mathbf{a}|}} & \mathbf{a} \subset \mathbf{b} \\ 0 & \mathbf{a} \not\subset \mathbf{b} \end{cases} \tag{34}$$

Notice that $P_{|\mathbf{a}||\mathbf{b}|}(t)$ is the entry in the reduced matrix that is here being used to generate multiple entries in the full matrix.

Appendix S1-O

Finding the matrix exponential Generally, we would diagonalize matrices so that we could take scalar exponentials of the eigenvalue matrix. However, because the patient–patient transmission rate matrix lacks a sufficient number of linearly independent eigenvectors, it is not possible to use this approach[11]. In this appendix, we use Jordan normal form to solve for the analytic equation for the patient–patient transmission rate matrix in terms of scalar (not matrix) exponentials. Because it is computationally expensive to factor large matrices into Jordan normal form, we then factor out γ so that factorization will not need to be performed repeatedly during the inference process, making optimization

over parameters computationally less intensive. In general, the transformation of a system to Jordan normal form is badly conditioned[5], but after matrix "compression" and factoring out γ, the patient–patient rate matrix contains integer values and is relatively small ($n = 13$, so the matrix has dimensions 14 by 14).

This solution allows us to calculate scalar exponentials of the Jordan matrix to find the matrix exponential. In our method, for a given hospital unit of size n, we factor out γ, the patient–patient transmission rate, leaving only integers within the matrix. We then perform Jordan factorization on the matrix using the symbolic toolbox in MATLAB so that no rounding error occurs. Because the result involves only scalar exponentiation, we vectorized over multiple matrices to speed calculation of results. (For larger matrices, matrix exponentials of the compressed rate matrix can be calculated using the built-in MATLAB function **expm** rather than scalar exponentiation using this Jordan form method, even though it may be slightly slower.)

Jordan form Let A be any $n \times n$ real matrix, and let t be a scalar. We wish to find the following exponential:

$$P = e^{At}$$

In general, a simple way to calculate the matrix exponential of At is to diagonalize At (if possible) and take the exponent of all the entries of the diagonal matrix Dt. Here, $Dt = V^{-1}(At)V$, where V is an $n \times n$ square matrix composed of column eigenvectors that correspond to the eigenvalues along the diagonal of Dt:

$$e^{At} = e^{V(Dt)V^{-1}} = V \begin{pmatrix} e^{\lambda_1 t} & & & \\ & e^{\lambda_2 t} & & \\ & & \ddots & \\ & & & e^{\lambda_n t} \end{pmatrix} V^{-1} \tag{35}$$

However, the matrix At cannot be diagonalized if there are an insufficient number of linearly independent eigenvectors. In this case, the matrix exponential of At can be calculated in an analogous manner using Jordan normal form.

Jordan normal form Any real square matrix A is similar to the block diagonal matrix J, that is, $J = V^{-1}AV$.

The Jordan matrix J has m block diagonals of the form

$$J = \begin{pmatrix} J_1 & & & \\ & J_2 & & \\ & & \ddots & \\ & & & J_m \end{pmatrix} \tag{36}$$

Each of the diagonal blocks is a bidiagonal matrix with the eigenvalue λ_k repeated down the diagonal and a sequence of 1's on the superdiagonal:

$$J_k = \begin{pmatrix} \lambda_k & 1 & & \\ & \lambda_k & 1 & \\ & & \ddots & 1 \\ & & & \lambda_k \end{pmatrix} \tag{37}$$

The eigenvector matrix V has m genuine eigenvectors and $n-m$ (non-genuine) generalized eigenvectors. Let each eigenvector $v_{k,j}$ be a column of V. Here, k is the index of a Jordan block J_k and its eigenvalue λ_k, and j is the index for eigenvectors associated with the eigenvalue. Each block has at least one genuine eigenvector ($j = 1$) and possible additional generalized eigenvectors ($j > 1$). By definition, the eigenvectors fulfill the following relationship:

$$Av_{k,j} = \begin{cases} \lambda_k v_{k,j} & j = 1 \\ \lambda_k v_{k,j} + v_{k,j-1} & j > 1 \end{cases} \tag{38}$$

Notice that the definition of generalized eigenvectors $v_{k,j}$ involves a sum with the previous eigenvector $v_{k,j-1}$.

Matrix exponential of the Jordan normal form We wish to find the matrix exponential for $P = e^{At}$ for the case in which A cannot be diagonalized because of an insufficient number of genuine eigenvectors. We use a method[2] that involves finding the Jordan normal form of the matrix At:

$$(At) = V(Jt)V^{-1} \tag{39}$$

Finding the matrix exponential for the Jordan normal form factorization involves taking matrix exponentials of the different Jordan blocks:

$$e^{At} = V \begin{pmatrix} e^{J_1 t} & & & \\ & e^{J_2 t} & & \\ & & \ddots & \\ & & & e^{J_m t} \end{pmatrix} V^{-1} \tag{40}$$

The exponential of each Jordan block with a particular eigenvalue λ is

$$e^{J_\lambda t} = e^{\lambda} \left(I + \frac{t}{1!}N + \frac{t^2}{2!}N^2 + \cdots + \frac{t^{n-1}}{(n-1)!}N^{n-1} \right) \tag{41}$$

where N is a square matrix of the same size as the Jordan block, with ones on the first superdiagonal and zeros everywhere else, that is, $N_{ij} = \delta_{i+1,j}$.

Written explicitly, the exponential of the product of a Jordan block J_λ and a scalar time t is an upper triangular matrix of form

$$e^{J_\lambda t} = e^{\lambda t} \begin{pmatrix} 1 & t/1! & & t^{n-1}/(n-1)! \\ & 1 & \ddots & \\ & & \ddots & t/1! \\ & & & 1 \end{pmatrix} \tag{42}$$

For example, the matrix exponential of a 2×2 Jordan block multiplied by a scalar time t is

$$J_\lambda t = e^{\lambda t} \begin{pmatrix} 1 & t \\ 0 & 1 \end{pmatrix} \tag{43}$$

Example matrix exponential for $n = 4$ beds As an example, we calculate the matrix exponential for the $n = 4$ bed case:

$$e^{At} = e^{(VJV^{-1})t} \tag{44}$$

$$= V \begin{pmatrix} e^{\lambda_1 t} & & & & \\ & e^{\lambda_2 t} & & & \\ & & e^{\lambda_3 t} & & \\ & & & e^{\lambda_4 t} & te^{\lambda_4 t} \\ & & & & e^{\lambda_4 t} \end{pmatrix} V^{-1} \tag{45}$$

Factoring out γ As it turns out, the most computationally expensive step for large matrices A is the process of factoring them into Jordan normal form. Although A can be quickly factored into Jordan normal form for small numbers of beds (such as the case where $n = 4$), it takes a tremendous amount of time when A is large ($n = 13$).

When trying to find maximum likelihood parameters, we must repeatedly exponentiate matrices containing different values of γ. In this section, we outline a shortcut by which we can exponentiate the Jordan matrix just once for a given number of beds n and reuse the factorization for multiple values of γ.

In the inference method of **Appendix S1-H**, the modified reduced matrix A is calculated from the reduced rate matrix R. We show an example of factorization for an $n = 4$ bed unit, but the principle holds for matrices for any number of beds:

$$A = \begin{pmatrix} 0 & 0 & & & \\ & -3\gamma & 3\gamma & & \\ & & -4\gamma & 4\gamma & \\ & & & -3\gamma & 3\gamma \\ & & & & 0 \end{pmatrix} \tag{46}$$

Factoring A into Jordan normal form is an expensive calculation and as previously described must be done for each instance of γ. However, γ can be factored out

of A as follows:

$$A = \begin{pmatrix} 0 & 0 & & & \\ & -3 & 3 & & \\ & & -4 & 4 & \\ & & & -3 & 3 \\ & & & & 0 \end{pmatrix} \gamma \tag{47}$$

Let

$$\hat{A} = \begin{pmatrix} 0 & 0 & & & \\ & -3 & 3 & & \\ & & -4 & 4 & \\ & & & -3 & 3 \\ & & & & 0 \end{pmatrix} \tag{48}$$

and $\tau = \gamma t$. Then

$$e^{At} = e^{\hat{A}\tau} = e^{(V\hat{J}V^{-1})\tau} \tag{49}$$

In general, $\hat{A}$ can be factored into $V\hat{J}V$ just once and the matrices saved. Thus, factoring out γ makes computation more efficient when calculations must be done for different values of A because we can simply factor $\hat{A}$ into Jordan normal form once. Then the resulting Jordan matrices V, $\hat{J}$, and V^{-1} can be reused for all computations regardless of the value of γ. Thus, Jordan factorization does not need to repeated for different values of γ.

Use of Jordan normal form is usually inadvisable because computation is unstable[11], as small perturbations or errors in calculation of the values of the eigenvalues can result in them being considered different. However, in this case, because $\hat{A}$ contains only integers, Jordan normal form factorization can be done symbolically and exactly.

References

1. Allen, L. J. S. (2011). *An Introduction to Stochastic Processes with Applications to Biology, Second Edition.* Hoboken: Taylor and Francis, 2nd ed. edition.

2. Bertolazzi, E. (2009). *Matrix exponential: Integration lectures for the Course: Numerical Methods for Dynamical System and Control.* Technical report, Tech. report, UNITN.

3. Cooper, B. S., Medley, G. F., Bradley, S. J., & Scott, G. M. (2008). An Augmented Data Method for the Analysis of Nosocomial Infection Data. *American Journal of Epidemiology*, 168(5), 548–557.

4. Dobrow, R. P. (2016a). Continuous-Time Markov Chains. In *Introduction to Stochastic Processes With R* (pp. 265–319). John Wiley & Sons, Inc.

5. Dooren, P. V. (2004). The basics of developing numerical algorithms. *IEEE Control Systems*, 24(1), 18–27.

6. Economou, A., Gómez-Corral, A., & López-García, M. (2015). A stochastic SIS epidemic model with heterogeneous contacts. *Physica A: Statistical Mechanics and its Applications*, 421, 78–97.

7. Gillespie, D. T. (1976). A general method for numerically simulating the stochastic time evolution of coupled chemical reactions. *Journal of Computational Physics*, 22(4), 403–434.

8. Gillespie, D. T. (1977). Exact stochastic simulation of coupled chemical reactions. *The Journal of Physical Chemistry*, 81(25), 2340–2361.

9. Gillespie, D. T. (1978). Monte Carlo simulation of random walks with residence time dependent transition probability rates. *Journal of Computational Physics*, 28(3), 395–407.

10. Kolmogoroff, A. (1931). Über die analytischen Methoden in der Wahrscheinlichkeitsrechnung. *Mathematische Annalen*, 104(1), 415–458.

11. Moler, C. & Van Loan, C. (2003). Nineteen Dubious Ways to Compute the Exponential of a Matrix, Twenty-Five Years Later. *SIAM Review*, 45(1), 3–49.

12. Shiryaev, A. N. (2012). Kolmogorov equation. In *Encyclopedia of Mathematics*. Kluwer Academic Publishers.

13. Simon, P. L., Taylor, M., & Kiss, I. Z. (2011). Exact epidemic models on graphs using graph-automorphism driven lumping. *Journal of Mathematical Biology*, 62(4), 479–508.
