# Supplementary material for: A mathematical model and inference method for bacterial colonization in hospital units applied to active surveillance data for carbapenem-resistant enterobacteriaceae
Source: PLoS One. 2020 Nov 12;15(11):e0231754. doi: 10.1371/journal.pone.0231754 (PMC7660488; doi:10.1371/journal.pone.0231754)
Supplement: S2 Appendix — (ZIP) [file pone.0231754.s002.zip › S2_Appendix.pdf]

S3 Supplemental Materials: Data Description

Data Description: A Mathematical Model and Inference Method for Bacterial Colonization in Hospital Units Applied to Active Surveillance Data for Carbapenem-Resistant Enterobacteriaceae

Karen M. Ong, Michael S. Phillips, Charles S. Peskin

The statistics for the rehabilitation unit alone are shown in **Table A**. The rehabilitation unit's average length of stay was 14.1 days. As shown in **Fig A**, the surveillance study had a duration of 417 days. For surveillance testing, after admission and at approximately weekly intervals thereafter, all patients in the hospital unit had perianal samples taken to test for *K. pneumoniae*, as previously described[6]. (Note that the dataset used was a subset from one of the two hospitals in the study.) Additionally, clinical microbiological tests, or test results from cultures ordered upon suspicion for infection, were included in the dataset. There were 936 surveillance tests and 12 clinical tests with 289 unique patients. Of these, 12/12 clinical tests were positive for CRE and 53/936 surveillance tests were positive for CRE. The overall number of positives was 65. Of these, 46 were *K. pneumoniae* positive. All events were time-stamped in the medical record to at least the day, hour, and minute.

	Symbol	Value	Units
average length of stay	$1/\beta$	14.1	d
turnover rate	β	0.071	d^{-1}
study length	t_Z	416.8	d

Table A. Statistics for the rehabilitation unit

Data Cleaning Of the 25 beds in the rehabilitation unit for which data was collected, only 13 consistently had patients, so we limited our analysis to the 13 beds (with 883 tests) in which patients were continually present, as shown in **Fig A**. (The omitted were either temporary or rarely used beds that remained empty for the majority of the surveillance study duration.)

We assumed that all entry times were entry/exit times and that if positives were followed by negatives for a given patient, that the negatives were false negatives as intestinal excretion may be intermittent[4] or may fall below the level needed for detection despite continuing colonization[8]. (Additional justification is found in "**Discussion**" section). We then extrapolated positives forward to the end of stay, after which we extrapolated any remaining negatives that did not follow positives on the same patient backwards to the beginning of stay, similar to the "data augmentation" performed by Cooper et. al[3]. Note that only 29 of 883 tests (3%) were potential false negatives, with 5 of these tests being highly likely to be false negatives, as they were found between positive tests within the same patient.

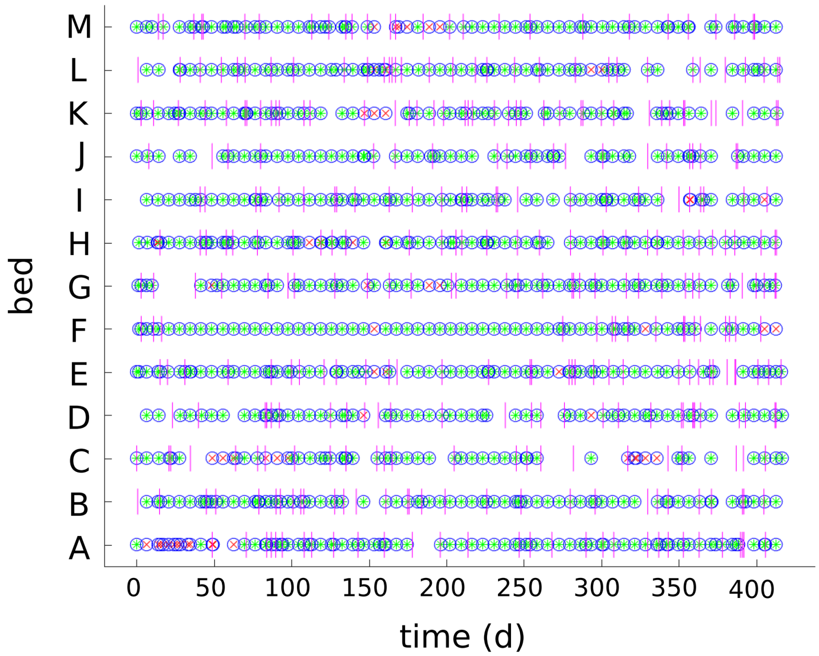

Figure A. Test results for the rehabilitation unit. Open circles indicate tests, green stars indicate negative test results for colonization, and red stars indicate positive test results for colonization. The magenta vertical bars indicate the times of entry/exit.

Although from a clinical standpoint, a negative following a positive culture result might be interpreted as the resolution of an infection, it would not necessarily mean the patient was decolonized, as tests have low sensitivity[2, 7] and colonization involves *asymptomatic* carriage of bacteria in the gastrointestinal tract[9]. We chose to assume "once colonized, always colonized" for several reasons: decolonization seems to occur on much longer timescales than hospital stays (months compared to days)[3, 4], intestinal excretion of bacteria is intermittent[4], tests have low sensitivity[2, 4], and few potential cases of false positive tests occurred in the rehabilitation data. As we had no ground truth for patient colonization with which to compare estimates of sensitivity, assuming "once positive, always positive" (such that negatives following positives are assumed to be false negatives) is a close approximation of CDC guidelines for decolonization, which suggest having greater than one negative test[2], or research guidelines suggested by Lubbert et al., which suggest having at least 4 consecutive negative rectal swabs or stool samples separated by at least weekly intervals, before declaring patient decolonization[4]. In our data, only 1 of 289 unique patients could qualify as being decolonized by the standard of Lubbert et al., although it should be noted that the patient had a long hospital stay

and thus might have a disproportionately large effect on parameter estimation. Although our dataset did not include antibiotic treatment, the case-control study on the same population by Swaminathan et al.[6] suggested that the odds of *acquiring* CRE increased by 4% for each day of antibiotic therapy rather than decreasing as might be expected if antibiotics result in decolonization. Additionally, the data was gathered prior to the widespread availability of highly effective treatements for CRE infection and colonization. Thus, it seems to be a reasonable simplification to assume that patients remain colonized during their hospitalizations. Incorporating decolonization into the model, especially in the light of newly available antimicrobials with more effective activity against CRE bacteria, is a valuable avenue for future research.

Distribution of Length of Stay The length of hospital stay is shown in **Fig B**. The length of stay in days was plotted against the natural logarithm of frequency (fraction of total patients with a particular length of stay). The time between events for a Poisson point process is described by the exponential distribution, a memoryless probability distribution in which β is the turnover time and $1/\beta$ describes the mean time between exit/entry events (e.g., the average length of stay)[5]. A plot of length of stay versus the natural logarithm of the frequency of occurrence should therefore be linear, as shown in **Fig B**, consistent with the assumption that the probability of patient exit/entry events follows a Poisson distribution. Thus in the reduced state model, the value of β can be estimated by taking the inverse of the average length of stay from the surveillance data. The assumption that patient turnover approximates a Poisson process is consistent with other studies regarding models for length of stay in the ICU[10] or after admission for surgery[1]. Both studies found generalized linear models with Poisson distributions and a logarithmic link to be among the most appropriate models. Here, generalized linear models are defined as extensions of the classical linear regression model that assume response variables have distributions from the exponential family and in which an arbitrary function of the response (the link function) varies linearly with the predictor[1].

References

1. Austin, P. C., Ghali, W. A., & Tu, J. V. (2003). A comparison of several regression models for analysing cost of CABG surgery. *Statistics in Medicine*, 22(17), 2799–2815.

2. Centers for Disease Control (2015). *Facility Guidance for Control of Carbapenem-resistant Enterobacteriaceae (CRE) â€“ November 2015 Update CRE Toolkit.* Technical report, Centers for Disease Control.

3. Cooper, B. S., Medley, G. F., Bradley, S. J., & Scott, G. M. (2008). An Augmented Data Method for the Analysis of Nosocomial Infection Data. *American Journal of Epidemiology*, 168(5), 548–557.

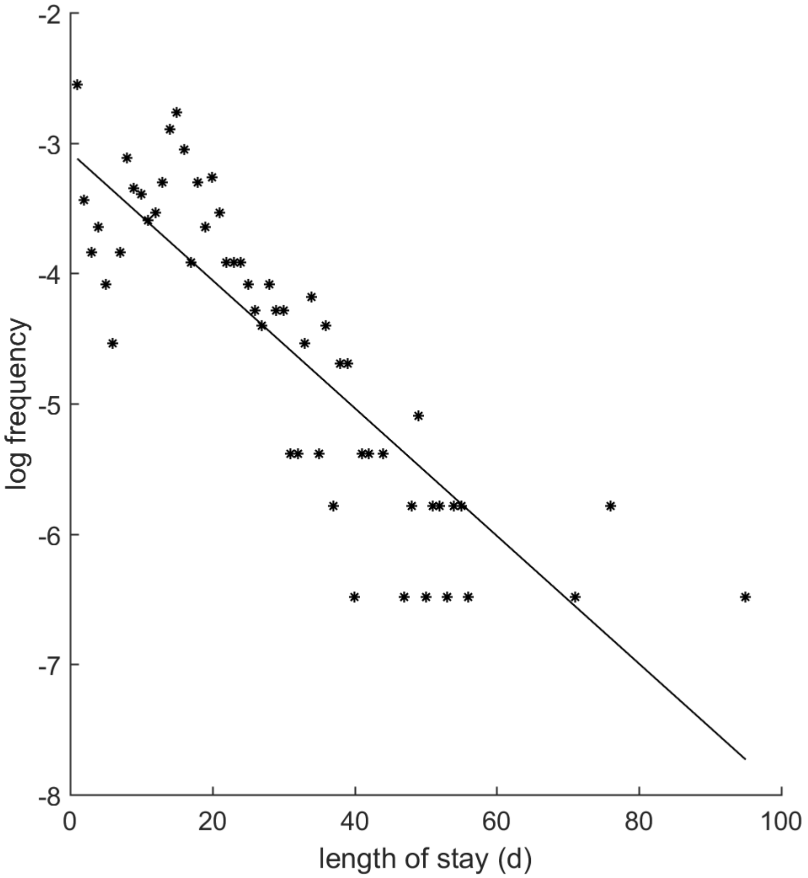

Figure B. Semi-log plot for distribution of length of stay in days versus the natural logarithm of frequency (fraction of the total patients with a particular length of stay). The black line shows the linear regression for the length of stay versus log frequency ($y = -3.0710 - 0.0490x$).

4. Lübbert, C., Becker-Rux, D., Rodloff, A., Laudi, S., Busch, T., Bartels, M., & Kaisers, U. (2014). Colonization of liver transplant recipients with KPC-producing Klebsiella pneumoniae is associated with high infection rates and excess mortality: A case-control analysis. *Infection*, 42(2), 309–316.

5. Sevast'yanov, BA (2002). Exponential distribution. In *Encyclopedia of Mathematics*. Kluwer Academic Publishers.

6. Swaminathan, M., Sharma, S., Blash, S. P., Patel, G., Banach, D. B., Phillips, M., LaBombardi, V., Anderson, K. F., Kitchel, B., Srinivasan, A., & Calfee, D. P. (2013). Prevalence and Risk Factors for Acquisition of Carbapenem-Resistant Enterobacteriaceae in the Setting of Endemicity. *Infection Control & Hospital Epidemiology*, 34(08), 809–817.

7. Snyder, G. M. & D'Agata, E. M. C. (2012). Diagnostic accuracy of surveillance cultures to detect gastrointestinal colonization with multidrug-resistant gram-negative bacteria. *American Journal of Infection Control*, 40(5), 474–476.

8. Temkin, E., Adler, A., Lerner, A., & Carmeli, Y. (2014). Carbapenem-resistant Enterobacteriaceae: biology, epidemiology, and management. *Annals of the New York Academy of Sciences*, 1323(1), 22–42.

9. Tischendorf, J., de Avila, R. A., & Safdar, N. (2016). Risk of infection following colonization with carbapenem-resistant Enterobactericeae: A systematic review. *American Journal of Infection Control*, 44(5), 539–543.

10. Verburg, I. W. M., de Keizer, N. F., de Jonge, E., & Peek, N. (2014). Comparison of Regression Methods for Modeling Intensive Care Length of Stay. *PLoS ONE*, 9(10).
