# Supplementary material for: A mathematical model and inference method for bacterial colonization in hospital units applied to active surveillance data for carbapenem-resistant enterobacteriaceae
Source: PLoS One. 2020 Nov 12;15(11):e0231754. doi: 10.1371/journal.pone.0231754 (PMC7660488; doi:10.1371/journal.pone.0231754)
Supplement: S1 Table — (ZIP) [file pone.0231754.s003.zip › S1_Table.pdf]

S2 Table: Comparison of Models

Study	Type of Model	State	Time	Mechanisms	Testing	Likelihood Calculation
[1]	Continuous-time ODE model of epidemic spread in population	reduced: S,E,I,Re	continuous	infected-susceptible transmission, immunity after infection, removal from susceptible population by recovery or death	n/a	compared calculated curve to number of deaths from plague on island of Bombay
[2]	Stochastic compartmental Markov model	reduced S,E,I,Re model	continuous	contact between infectious and susceptible patients	incomplete data	MCMC with reversible jump methodology and Metropolis-Hasting sampler
[3]	Deterministic ODE compartmental model of VRE nosocomial transmission	reduced: number UC and C patients, UC and C HCWs	continuous	HCWs act as vectors between C and UC patients, antibiotic selection pressure, cohorting, handwashing	n/a	parameters estimated directly from surveillance data (not fitted), Monte Carlo simulations performed and compared with original results
[4]	Markov model of longitudinal data about pneumococcal carriage in families	full: track UC/C status for each family member, serotype, initial colonization status	discrete-time	in-family vs. community-acquired transmission	Bayesian data augmentation method for 86% data observations, missing data assumed to be missing at random; Bayesian data augmentation	Metropolis-Hastings sampling of posterior distribution with reversible jump MCMC extension
[5]	Continuous-time Markov Model for VRE and *Pseudomonas aeruginosa* transmission within ICUs	reduced: number UC/C patients (prevalence)	continuous	exogenous (cross-transmission) vs. endogenous colonization (from antibiotic pressure), pre-existing colonization	surveillance data, time-series of colonized patient compared with genotyping gold standard	maximum likelihood fit, calculates stationary distributions, matrix exponentiation
[6]	Continuous-time mechanistic structured hidden Markov model for MRSA, VRE, RGNR in ICUs	reduced state SI	continuous	patient–patient transmission model (mass action assumption, assesses Greenwood assumption of saturating infectivity), pre-existing colonization	clinical cultures only (x 40 mo, not whole-ward surveillance swabs)	matrix exponential, likelihood maximization, simulation for ranges, stationary distribution
[7]	Continuous-time hidden Markov model of VRE	SI reduced with migration	continuous	sporadic (pre-existing, exogenous non-ward sources, endogenous colonization) and cross-transmission	VRE weekly prevalence data	MCMC Bayesian Framework, Baum's recursion formula
[8]	Stochastic epidemic model for transmission of asymptomatic pathogens (MRSA) within a ICU with isolation	S, C, Is+C, Re	discrete time	C in ward from non-isolated C, isolated C; C in ward from random source (0.0103 transmissions per day, 0.047 mean importation probability); C prior to admission	perfect compliance for observations, imperfect sensitivity, perfect specificity; data has admission, positive swab, isolation, discharge times; augmented data with unobserved colonization times and final patient states; assess of goodness of fit by comparing observed and replicated data	reversible-jump MCMC within Bayesian framework to allow for imperfect sensitivity, used non-aggregated patient level data in MCMC
[9]	Markov model of CRE that allows for changing state space and incorporates culture results	detailed state of unit/ prevalence UC/C/unknown	discrete (smallest unit of time is 1 day)	exogenous / endogenous, pre-existing	partial with extrapolation	maximum-likelihood estimates with confidence intervals using matrix method
[10]	Stochastic VRE model variants (including multiple hospital admissions)	reduced state UC/C	discrete observations, continuous-time(?)	patient–patient transmission, pre-existing colonization, colonization between hospital visits	incomplete, extrapolated, data augmented with transmissions, imperfect sensitivity	MCMC and Bayesian inference
[11]	VRE surveillance model that uses Martingale-based methodology	reduced model UC/ C / Re	continuous	non-constant patient–patient transmission (Reed-Frost and proportionate mixing/true mass action assumptions), pre-existing colonization	perfect observations assumed	martingale methods for nonparametric estimates of transmission rate
[12]	Multi-variate Markov model for MRSA transmission	reduced UC/C Pts, UC/C HCWs (as function of C pts) **incidence** data	continuous	HCW-patient and patient-HCW transmission, pre-existing colonization, decolonization of HCW by handwashing	imperfect testing, daily/weekly incidence	maximum likelihood using **matrix exponentials**
[13]	15-parameter Bayesian stochastic spatiotemporal compartment model of *C. difficile* with multiple rooms	SIR variant: S+UC, S+C, Im-Abx, Im+Abx, Tox+	continuous-time(?)	colonized from Tox+ or S+C patient in same room, C or TP in different room, background environmental sources; arrive into any state	data augmentation, 30-bed geriatric units	Bayesian framework with MCMC methods, maximum likelihood for some parameters
[14]	Stochastic 5-parameter MRSA model of isolation in 8 ICUs (based on [8])	UC/(C+Is)/(C+non-Is), compared linear vs. no-background vs. nonlinear transmission risk	discrete-time	transmission from background, C+non-Is, and C+Is pts; pre-existing prevalence	estimates swab sensitivity, assumes perfect specificity	Bayesian framework, data-augmented (unobserved colonization times included as additional parameters) MCMC to estimate mean percent colonized patient-days attributed to undetected carriers from weekly screening cultures
[15]	Stochastic 5-parameter MRSA model of hospital general wards with isolation and decolonization	UC/(C+Is)/(C+non-Is), linear transmission risk with C and UC pts	discrete-time	transmission from background, C+non-Is, and C+Is pts; pre-existing prevalence	estimates swab sensitivity, assumes perfect specificity	MCMC
[16]	Stochastic 5-parameter VRE model of isolation in 8 ICUs	UC/(C+Is)/(C+non-Is), compared linear vs. no-background (better for medical units) vs. nonlinear transmission risk (better for surgical units)	continuous-time	transmission from background, C+non-Is, and C+Is pts; pre-existing prevalence	estimates swab sensitivity, assumes perfect specificity	data-augmented MCMC tracks individual patient statuses and approximates colonization times
[17]	Generalized stochastic multi-compartmental SIS model for nosocomial spread within hospital settings	generic agents could include pts, HCWs, multiple states	continuous-time	5 examples including hand hygiene, environmental cleaning, super-spreaders, spatial layout	finds probability distribution of basic reproduction number	matrix calculation for reproduction number

VRE = vancomycin-resistant enterococci, RGNR = third-generation cephalosporin-resistant gram-negative rods; MRSA = methacillin-resistant *Staphylococcus aureus*; ODE = ordinary differential equation; S = susceptible, I = infected, Is = isolated, R = recovered, Re = removed, E = latent, UC = uncolonized, C = colonized, HCW = healthcare worker; ML = maximum likelihood, MCMC = Markov chain Monte Carlo; exogenous colonization = cross-transmission, endogenous colonization = colonization from antibiotic administration and selection of strains; S+UC = susceptible and uncolonized, S+C = susceptible and colonized, Im-Abx = immune without antibiotics, Im+Abx = immune with antibiotics, Tox+ = toxin positive, Is = Isolated.

References

1. Kermack, W. O. & McKendrick, A. G. (1927). A Contribution to the Mathematical Theory of Epidemics. *Proceedings of the Royal Society of London A: Mathematical, Physical and Engineering Sciences*, 115(772), 700–721.

2. Gibson, G. J. & Renshaw, E. (1998). Estimating parameters in stochastic compartmental models using Markov chain methods. *Mathematical Medicine and Biology*, 15(1), 19–40.

3. Austin, D. J., Bonten, M. J. M., Weinstein, R. A., Slaughter, S., & Anderson, R. M. (1999). Vancomycin-resistant enterococci in intensive-care hospital settings: Transmission dynamics, persistence, and the impact of infection control programs. *Proceedings of the National Academy of Sciences*, 96(12), 6908–6913.

4. Auranen, K., Arjas, E., Leino, T., & Takala, A. K. (2000). Transmission of Pneumococcal Carriage in Families: A Latent Markov Process Model for Binary Longitudinal Data. *Journal of the American Statistical Association*, 95(452), 1044–1053.

5. Pelupessy, I., Bonten, M. J. M., & Diekmann, O. (2002). How to assess the relative importance of different colonization routes of pathogens within hospital settings. *Proceedings of the National Academy of Sciences*, 99(8), 5601–5605.

6. Cooper, B. & Lipsitch, M. (2004). The analysis of hospital infection data using hidden Markov models. *Biostatistics*, 5(2), 223–237.

7. McBryde, E., Pettitt, A., Cooper, B., & McElwain, D. (2007). Characterizing an outbreak of vancomycin-resistant enterococci using hidden Markov models. *Journal of the Royal Society Interface*, 4(15), 745–754.

8. Forrester, M. L., Pettitt, A. N., & Gibson, G. J. (2007). Bayesian inference of hospital-acquired infectious diseases and control measures given imperfect surveillance data. *Biostatistics*, 8(2), 383–401.

9. Bootsma, M. C. J., Bonten, M. J. M., Nijssen, S., Fluit, A. C., & Diekmann, O. (2007). An Algorithm to Estimate the Importance of Bacterial Acquisition Routes in Hospital Settings. *American Journal of Epidemiology*, 166(7), 841–851.

10. Cooper, B. S., Medley, G. F., Bradley, S. J., & Scott, G. M. (2008). An Augmented Data Method for the Analysis of Nosocomial Infection Data. *American Journal of Epidemiology*, 168(5), 548–557.

11. Wolkewitz, M., Dettenkofer, M., Bertz, H., Schumacher, M., & Huebner, J. (2008). Statistical epidemic modeling with hospital outbreak data. *Statistics in Medicine*, 27(30), 6522–6531.

12. Drovandi, C. C. & Pettitt, A. N. (2008). Multivariate Markov Process Models for the Transmission of Methicillin-Resistant Staphylococcus Aureus in a Hospital Ward. *Biometrics*, 64(3), 851–859.

13. Starr, J. M., Campbell, A., Renshaw, E., Poxton, I. R., & Gibson, G. J. (2009). Spatio-temporal stochastic modelling of Clostridium difficile. *Journal of Hospital Infection*, 71(1), 49–56.

14. Kypraios, T., O'Neill, P. D., Huang, S. S., Rifas-Shiman, S. L., & Cooper, B. S. (2010). Assessing the role of undetected colonization and isolation precautions in reducing Methicillin-Resistant Staphylococcus aureustransmission in intensive care units. *BMC Infectious Diseases*, 10(1), 29.

15. Worby, C. J., Jeyaratnam, D., Robotham, J. V., Kypraios, T., O'Neill, P. D., De Angelis, D., French, G., & Cooper, B. S. (2013). Estimating the Effectiveness of Isolation and Decolonization Measures in Reducing Transmission of Methicillin-resistant Staphylococcus aureus in Hospital General Wards. *American Journal of Epidemiology*, 177(11), 1306–1313.

16. Wei, Y., Kypraios, T., O'Neill, P. D., Huang, S. S., Rifas-Shiman, S. L., & Cooper, B. S. (2016). Evaluating hospital infection control measures for antimicrobial-resistant pathogens using stochastic transmission models: Application to vancomycin-resistant enterococci in intensive care units:. *Statistical Methods in Medical Research.*

17. López-García, M. & Kypraios, T. (2018). A unified stochastic modelling framework for the spread of nosocomial infections. *Journal of The Royal Society Interface*, 15(143), 20180060.
